# Stability analysis of roadbed under flood scouring

**DOI:** 10.1038/s41598-024-54765-8

**Published:** 2024-02-21

**Authors:** Rui Wang, Hongmei Tang, Fuchuan Zhou

**Affiliations:** 1 Institute of Geotechnical Engineering, Chongqing Jiaotong University, 66 Xuefu Road, Nan’an District, Chongqing, 400074 People’s Republic of China; 2 Chongqing Jiaotong University, 66 Xuefu Road, Nan’an District, Chongqing, 400074 People’s Republic of China; 3 Chongqing Jianzhu College, Chongqing, 400072 People’s Republic of China; 4 Chongqing Vocational Institute of Engineering, Chongqing, 402260 People’s Republic of China; 5 School of Civil Engineering, Chongqing University, Chongqing, 400030 People’s Republic of China

**Keywords:** Flood, Soil roadbed along the river, Water erosion evolution, Stability safety factor, Natural hazards, Engineering

## Abstract

Soil roadbed along the river suffers from water erosion at the bottom and collapse at the top under flood scouring, which leads to the suspension of upper pavement slab. In order to ensure the safety of soil roadbed along the river, this study explored the development mechanism of soil roadbed damage by flood in actual cases, and proposed the evolution process of instability under roadbed scouring. The stability law of roadbed along the river under flood scouring was analyzed, and the stability safety factor was corrected to analyze the sensitivity of water depth, flow rate, river bending angle and stability safety factor K in working conditions. The sensitivity of width and height of soil roadbed after flood scouring to water depth, flow velocity, river bending angle was investigated. Moreover, numerical simulation was carried out to determine the displacement nephogram and maximum shear stress nephogram of soil roadbed along the river under the conditions of road surface and roadbed load, vehicle loading or constant change of water depth. By comparing the above theories and engineering cases, the water damage mechanism of soil roadbed along the river was further verified.

## Introduction

The complex topography restricts the planning and design of mountain roads. Take central and western China for example, research data and field survey results show that there are a large number of roads along the river in the western mountainous areas. With the promotion of the West China Development strategy, there have been huge demands for new roads along the river. Roads along the river in mountainous areas are mostly semi-filled and semi-dug roadbed, which is vulnerable to long-term scouring by rivers and direct impact by floods or mudslides, resulting in disasters such as roadbed damage, pavement suspension or fracture. This seriously affects the traffic operation of roads in mountainous areas and poses great security risks to local economy and people's lives and property. At present, the flood damage of highway along the river has not been cured, instead, it has become more and more frequent. Therefore, it is of great significance to study the evolution law of water erosion damage of roadbed along the river and implement immediate protection.

Among the studies of roadbed instability due to river water erosion, Chen^1^ solved the formula of critical water erosion groove radius of roadbed gap according to the limit equilibrium theory to determine the gap formation mechanism. Zhao et al.^2^ carried out numerical simulation analysis to simulate the change of roadbed along river under flood scouring. According to Liang Dan et al.^3^, the erosion and waterout mechanism of roadbed along the river is essentially the interaction process between the erosion resistance of roadbed rock and soil and the impact force of roadbed near the wall. Kawajiri et al.^4^ explored the ground erosion and roadbed changes supporting piers by carrying out large-scale open channel model tests. Kurdistani et al.^5^ studied the effects of structural geometry, riverbed material and river hydraulic conditions on the downstream scour pattern of stepped weir. Campbell et al.^6^ investigated the integrated geophysical technology for underwater detection of bridge foundation related scour and erodible scour filler. Daneshfaraz et al.^7^ conducted numerical simulation of river scouring pile groups. Choudhary et al.^8^ developed ANFIS- and GEP-based model for prediction of scour depth around bridge pier.

Based on the above analysis, it can be seen that the studies of erosion of roadbed along the river in many countries focus on soil slope erosion test (Khan et al.^9^, Guan et al.^10^), numerical simulation (Nguyen et al.^11^) and result analysis (Sharafati et al.^12^). Through the evolution of water erosion groove formed by flood scouring roadbed toe, this study analyzed the concrete mechanical instability evolution process of roadbed scouring in operation stage, and explored the sensitivity law of roadbed stability safety factor and related factors. It is an urgent problem to be solved for the concrete evolution process of instability of soil roadbed scouring along the river under the continuous action of flood, gravity and vehicle load and the suspension of the upper pavement slab.

## Scouring characteristics of roadbed along the river

The studies of scouring mechanism of soil roadbed along the river focus on the following four aspects: the evolution mechanism of soil roadbed scouring instability, the main controlling factors of soil roadbed scouring, the easily damaged sections of soil roadbed scouring and the disaster time. First, the scouring process of soil roadbed along the river begins with sediment incipient motion. It is considered that the sediment particles or slope soil particles at the slope toe are washed away by river after sediment incipient motion. The porosity of the slope soil increases, the soil roadbed infiltrates, and the slope strength index decreases, which result in the instability and collapse of soil roadbed slope, the scouring damage of soil roadbed and the suspension or collapse of pavement slab. Second, there are three main factors influencing soil roadbed scouring along the river: soil roadbed conditions, water flow conditions and watershed boundary conditions. Third, the most unfavorable scouring position of soil roadbed along the river is at the concave bank of river bend. Fourth, the damage of soil roadbed along the river mainly occurs at the flood scouring stage at the soil roadbed side.

**Figure 1:**
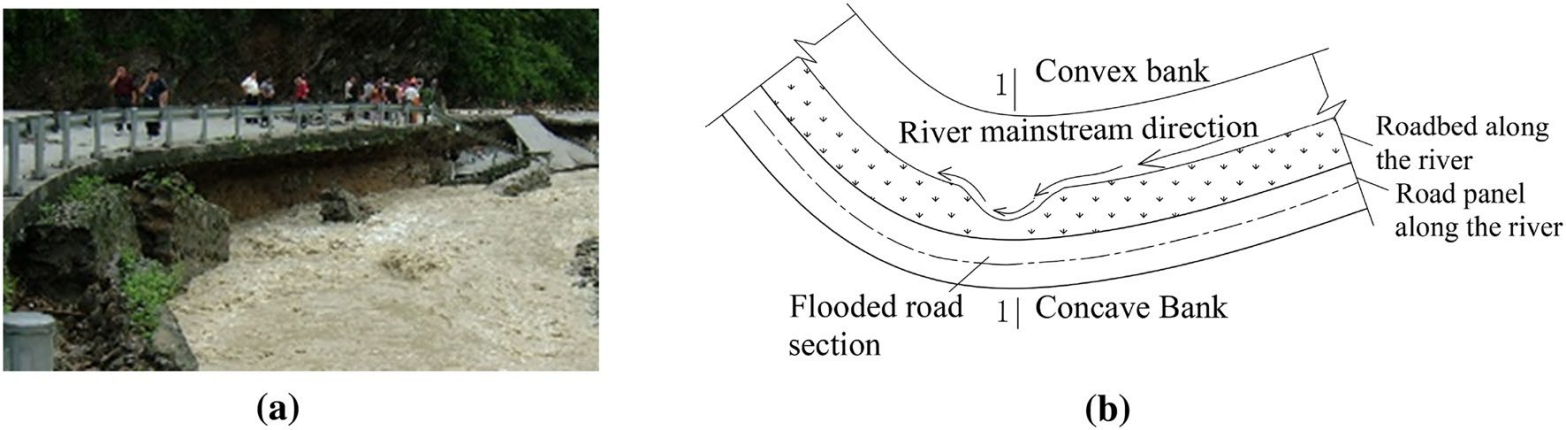
Erosion of roadbed along the river. (**a**) Water damaged roadbed along the line, (**b**) River plan.

**Figure 2:**
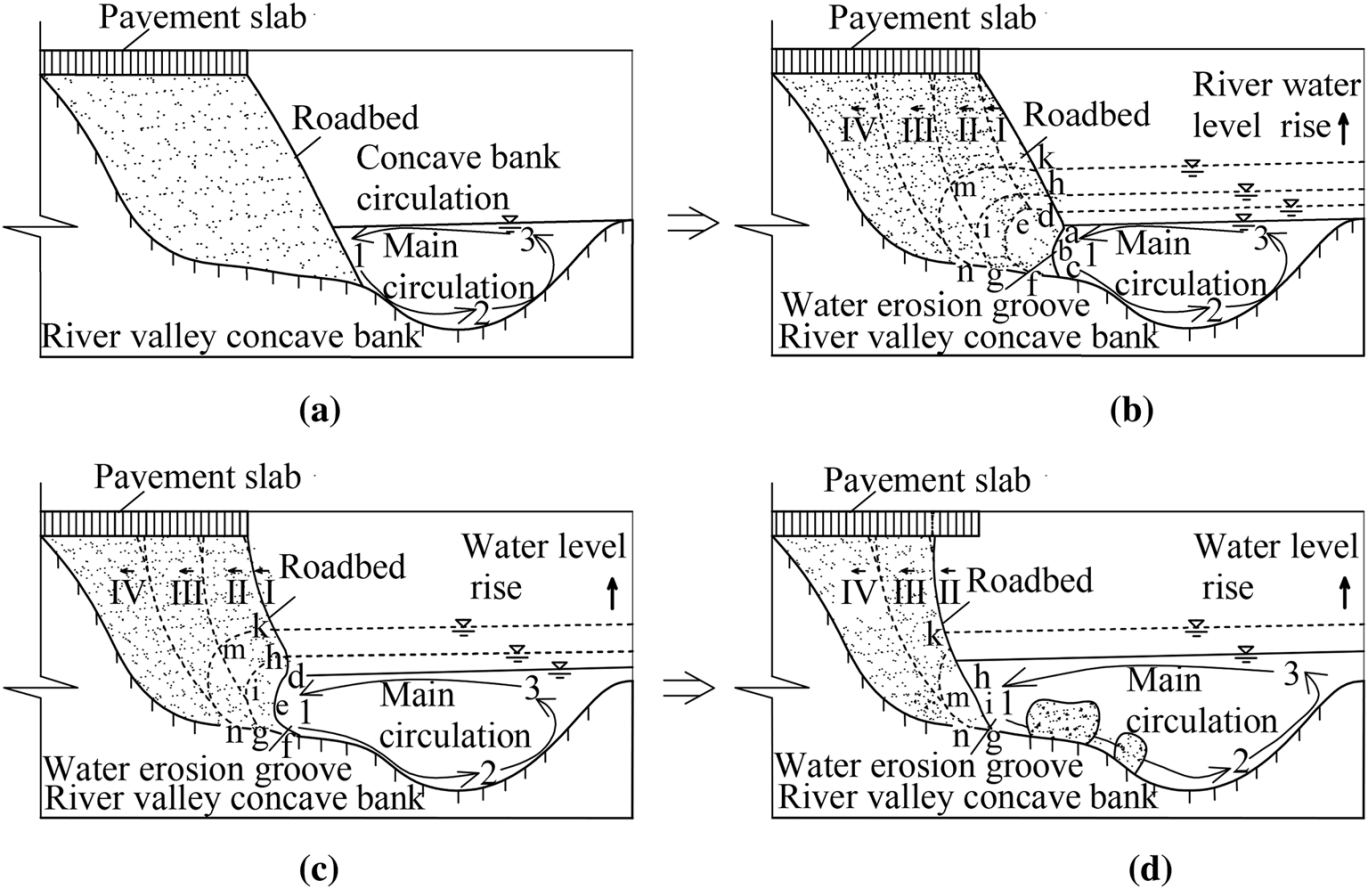
Evolution process of suspended pavement slab. (**a**) Slope toe of roadbed along the river eroded by concave bank, (**b**) Water erosion groove of roadbed along the river, (**c**) Roadbed soil slides along the dangerous sliding surface, (**d**) Suspension of pavement slab caused by the sliding instability of roadbed soil.

As shown in Fig. 1a, flood scouring leads to the lack of soil roadbed at the concave bank and obvious suspension of pavement slab. Figure 1b is a plan view of roadbed washout at the concave bank, in which Sect. 1–1 shows four stages of soil roadbed lack under flood scouring, as shown in Fig. 2.

(1) Flood scouring stage: In flood season, the velocity and discharge of water flow increase rapidly, and there are a large amount of sediment and gravel in flood, which repeatedly washes away the soil roadbed slope toe. After the scouring action of the main flood circulation 123 reaches the critical state of sediment incipient motion, the soil at the soil roadbed slope toe will be eroded by scouring water, as shown in Fig. 2a.

(2) Formation and expansion stage of water erosion groove: Under normal river conditions, the strength of soil roadbed will gradually decrease after long-term immersion of the soil roadbed slope toe, and there will be potentially dangerous sliding surfaces in soil roadbed soil. Assuming that the rising flood in the flood season constantly washes away the toe of the water-eroded soil roadbed, the initial arc-shaped water-eroded groove a-b-c is locally derived from the soil roadbed. Four potential sliding surfaces I, II, III and IV are developed inside the roadbed, as shown in Fig. 2b.

(3) Soil roadbed sliding instability stage: After the soil roadbed is eroded by flood, the soil at the top of the water erosion groove will expand from a-b-c to def with the rise of flood level under the action of water flow and gravity. In the meanwhile, the upper soil will slide along the sliding surface I. Water erosion groove expands to h-i-g under continous flood scouring, and the upper soil slides along the sliding surface II, as shown in Fig. 2c.

(4) Suspension and instability stage of pavement slab: the water erosion groove continuously expands to the vicinity of the limit state k-m–n. With the increase of the suspension ratio, when the pavement slab reaches the critical state of fracture and failure, fracture and failure will occur, as shown in Fig. 2d.

## Washout development mechanism of roadbed along the river

### Stress analysis of soil erosion at the roadbed slope toe

**Figure 3:**
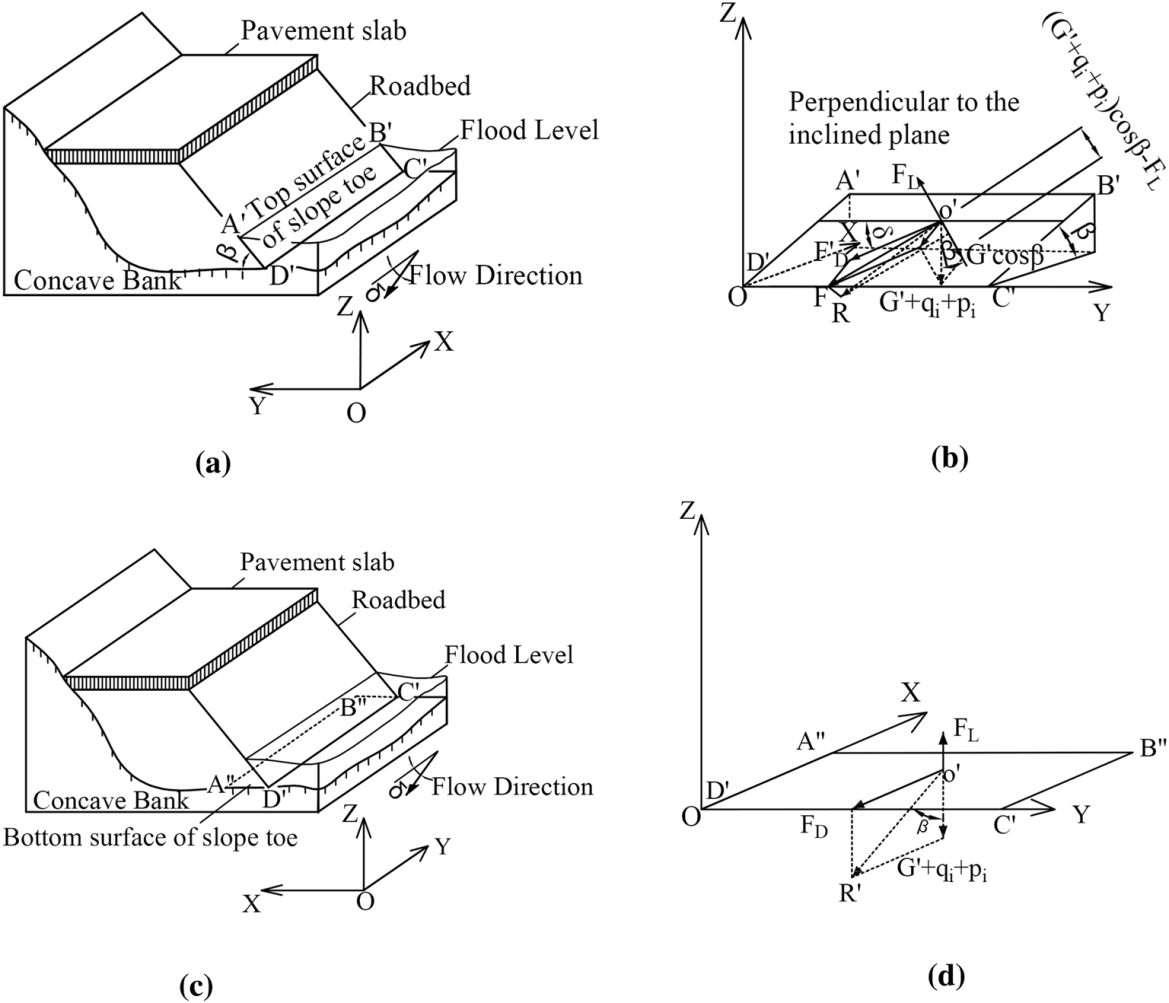
Schematic diagram of stress analysis of sediment particles on roadbed slope. (**a**) Schematic diagram of the top surface of slope toe, (**b**) Schematic diagram of stress on the top surface of slope toe, (**c**) Schematic diagram of the bottom surface of slope toe, (**d**) Schematic diagram of stress on the bottom surface of slope toe.

Soil roadbed along the river is often washed by the river, and the sliding force on the slope surface caused by river impact and gravity at the soil roadbed slope toe is related to soil roadbed slope. The stress at the soil roadbed slope toe in Fig. 1a is analyzed, as shown in Fig. 3.

#### Load analysis of soil at the top of roadbed slope toe

Assume that the inclination angle of roadbed slope is β,the included angle between water flow and the horizontal axis of roadbed slope is δ, the impact drag force is F_D_′, the lifting force is F_L_, the weight of soil is G′, the vehicle load is p_i_, the weight of pavement slab is q_i_, and the viscous force of sediment is C_k_, as shown in Fig. 3a,b. The resultant force acting on the slope surface together is as below:

1 $$ F = \sqrt {\left[ {F_{D}{^{\prime }} \sin \delta + \left( {G{^{\prime}} + p_{i} + q_{i} } \right)\sin \beta } \right]^{2} + F_{D}{^{\prime 2}} \cos^{2} \delta } $$

The corresponding anti-sliding force is:

2 $$ F_{R} = C_{k} + \left[ {\left( {G^{\prime} + p_{i} + q_{i} } \right)\cos \beta - F_{L} } \right]\tan \beta $$

The starting conditions of sediment at the roadbed slope toe are as follows

3 $$ \sqrt {\left[ {F_{D}{^{\prime }} \sin \delta + \left( {G^{\prime} + p_{i} + q_{i} } \right)\sin \beta } \right]^{2} + F_{D}{^{\prime 2}} \cos^{2} \delta } \ge C_{k} + \left[ {\left( {G^{\prime} + p_{i} + q_{i} } \right)\cos \beta - F_{L} } \right]\tan \beta $$

According to the above formula, the impact drag force of roadbed slope toe is as below:

4 $$ F^{\prime}_{D} = - \left( {G^{\prime} + p_{i} + q_{i} } \right)\sin \delta \sin \beta + \sqrt {\left[ {C_{k} + \left( {\left( {G^{\prime} + p_{i} + q_{i} } \right)\cos \beta - F_{L} } \right)\tan \beta } \right]^{2} - \left( {G^{\prime} + p_{i} + q_{i} } \right)^{2} \sin \beta \cos^{2} \delta } $$

#### Analysis of soil load on the bottom surface of roadbed slope toe

For the bottom surface of soil roadbed slope toe, as shown in Fig. 3c,d, the corresponding sliding resistance is as follows:

5 $$ F_{R} = C_{k} + \left( {G^{\prime} + p_{i} + q_{i} - F_{L} } \right)\tan \beta $$

The impact drag force of soil roadbed slope plane is F_D_, and the condition of sediment incipient motion is:

6 $$ F_{D} \ge F_{R} = C_{k} + \left( {G^{\prime} + p_{i} + q_{i} - F_{L} } \right)\tan \beta $$

Set τ_c_ as the incipience drag force of soil roadbed toe bottom surface and τ_c_′ as the incipience drag force of roadbed toe top surface.

7 $$ \frac{{\tau^{\prime}_{c} }}{{\tau_{c} }} = \frac{{F^{\prime}_{D} }}{{F_{D} }} $$

Substitute formulas (4)–(6) into formula (7)

8 $$ \begin{gathered} \frac{{\tau^{\prime}_{c} }}{{\tau_{c} }} = \sqrt {\left[ {1 - \left( {1 - \frac{{C_{k} }}{{F_{D} }} + \frac{{F_{L} }}{{F_{D} }}\tan \beta } \right)\left( {1 - \cos \beta } \right)} \right]^{2} - \left[ {1 - \frac{{C_{k} }}{{F_{D} }} + \frac{{F_{L} }}{{F_{D} }}\tan \beta } \right]^{2} \cos^{2} \beta \cos^{2} \delta } \\ - \left[ {\left( {1 - \frac{{C_{K} }}{{F_{D} }}} \right)\cos \beta \sin \delta + \frac{{F_{L} }}{{F_{D} }}\sin \beta \sin \delta } \right] \\ \end{gathered} $$

When δ = 0, that is, the water flow is orthogonal to the inclined direction of soil roadbed slope toe, the sediment incipience conditions of soil roadbed slope along the river are as follows:

9 $$ \frac{{\tau^{\prime}_{c} }}{{\tau_{c} }} = \sqrt {\left[ {1 - \left( {1 - \frac{{C_{k} }}{{F_{D} }} + \frac{{F_{L} }}{{F_{D} }}\tan \beta } \right)\left( {1 - \cos \beta } \right)} \right]^{2} - \left[ {1 - \frac{{C_{k} }}{{F_{D} }} + \frac{{F_{L} }}{{F_{D} }}\tan \beta } \right]^{2} \cos^{2} \beta } $$

$F_{L} = 0$, and the lifting force is ignored:

10 $$ \frac{{\tau^{\prime}_{c} }}{{\tau_{c} }} = \sqrt {\left[ {1 - \left( {1 - \frac{{C_{k} }}{{F_{D} }}} \right)\left( {1 - \cos \beta } \right)} \right]^{2} - \left[ {1 - \frac{{C_{k} }}{{F_{D} }}} \right]^{2} \cos^{2} \beta } $$

According to Formulas (6)–(10), under the same water flow, the shear force required for sediment incipience on the soil roadbed slope is less than that on the bottom surface and sediment incipience is more likely to occur on the top surface of the slope toe. Therefore, the river water erosion drives the top surface of the soil roadbed slope toe earlier and easier compared with the bottom surface.

### Stress analysis of soil falling on the upper part of roadbed water erosion groove

After the water erosion groove at the bottom of soil roadbed soil is missing, the stress on the upper soil changes, and the soil gradually falls. In videos of flood scouring the soil roadbed along the river, the soil on the upper part of the soil roadbed is irregular (the dotted lines in Fig. 2b–d), falls vertically to the bottom and is washed away by the flood, and the rest is deposited.

**Figure 4:**
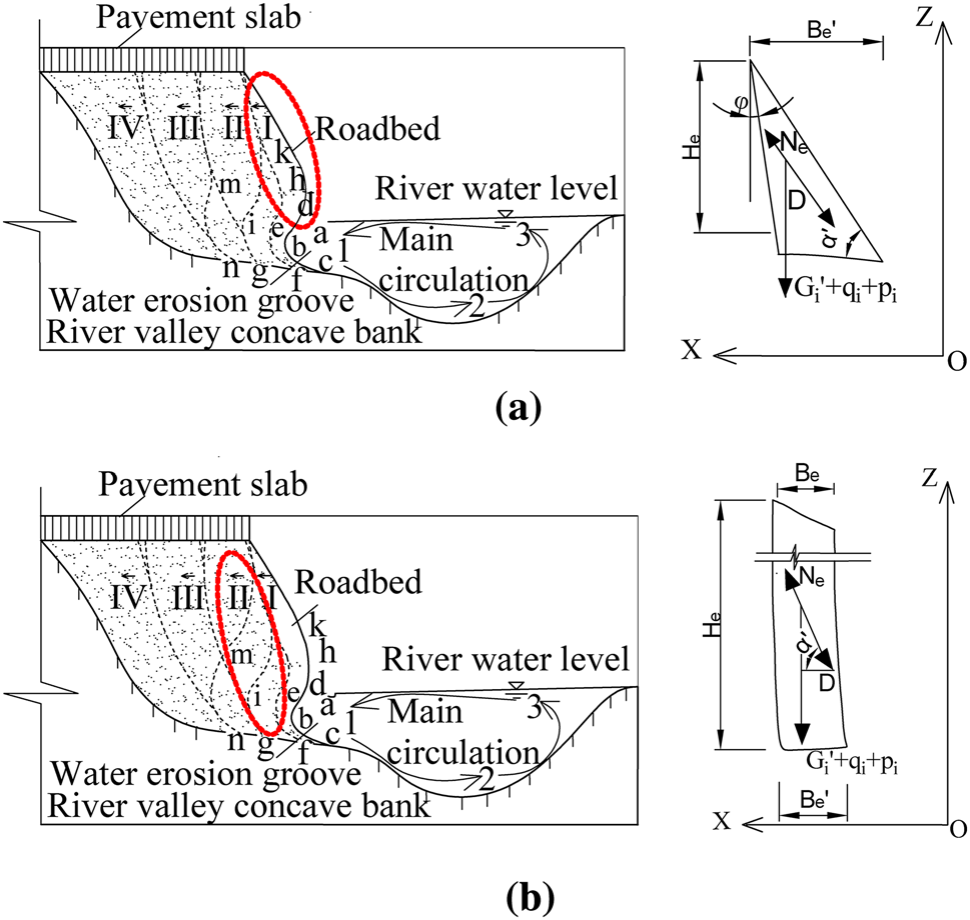
Schematic diagram of stress analysis of soil at the top of roadbed. (**a**) Stress analysis of outer soil falling at the top of roadbed, (**b**) Stress analysis of inner soil falling at the top of roadbed.

Figure 4a,b analyze the stress. Assume that the horizontal dip angle of the upper soil roadbed soil is α′, the weight of the soil is G_i_′, the vehicle load is p_i_, the weight of the pavement slab is q_i_, the shear resistance of the soil is N_e_, the vertical dip angle is φ, the width of the upper part is B_e_, and the height is H_e_:

Stress analysis is carried out for the outer and inner soil at the top of roadbed:

11 $$ F = G_{i}^{\prime } + p_{i} + q_{i} + \left( {D - N_{e} } \right)\sin \alpha $$

The main reason for the falling of flaky soil at the top of roadbed is that the bottom part of is eroded by water, softened, slumped and washed away, which will lose its supporting function to the upper flaky soil. When the water erosion and scouring depth of the bottom water erosion groove reaches a certain size before the upper soil falls every time, the upper flaky soil will fall after overcoming the shear resistance between soil particles under the action of gravity. In this process, the depth dimension of the bottom water erosion groove is equivalent to the width Be′ dimension of the upper flaky soil. In addition, due to the environmental factors such as geography, topography, rainfall and seepage, seepage force will be formed, thus affecting the upper soil. Therefore, the flake size of the falling flaky soil is related to the density, material properties, water erosion depth, scouring depth, flake height of the soil and the hanging width, and most of the falling parts are flake structures.

To sum up, from the stress analysis in Sects. 3.1 and 3.2, it can be found that the falling position of the roadbed soil along the river caused by water erosion is mainly determined by the water erosion groove of the bottom soil. Therefore, the change of the water erosion groove angle of the soil roadbed will affect not only the integrity and missing direction of the whole soil roadbed, but also the proportion and position of the upper pavement slab suspension.

## Stability analysis of roadbed washout along the river

### Overall stability analysis of roadbed with water erosion groove under the gravity of vehicle and pavement slab

**Figure 5:**
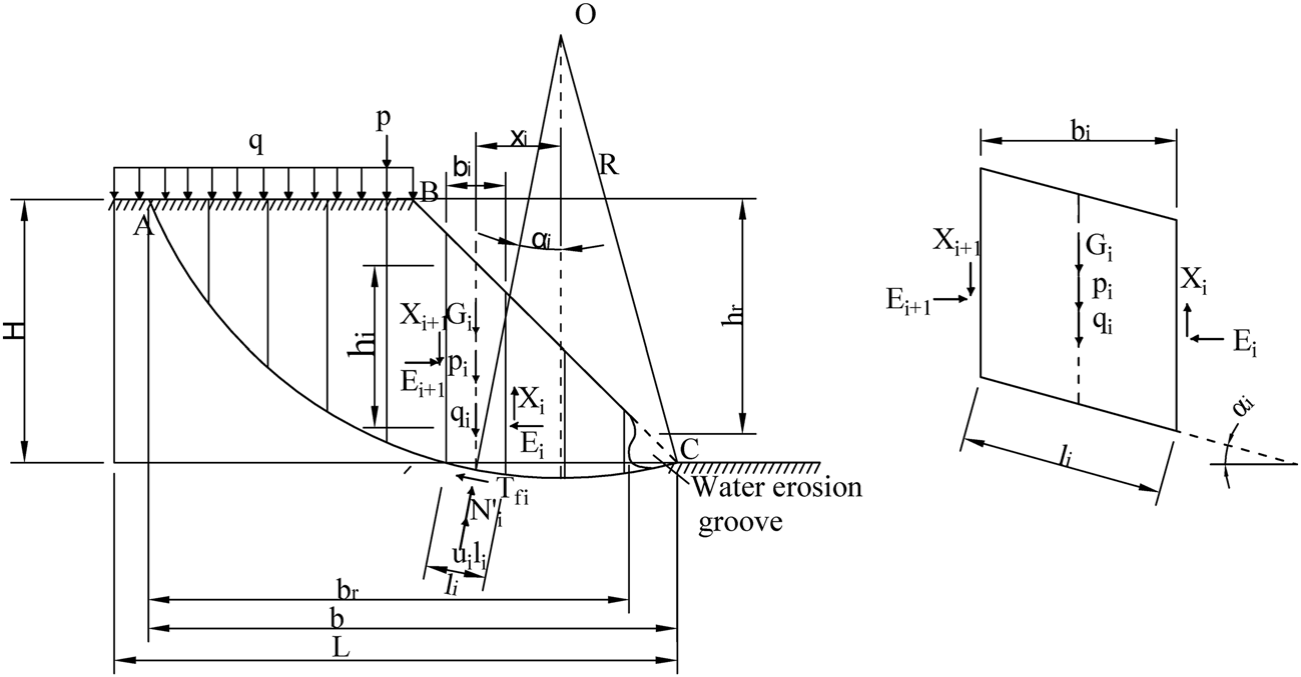
Diagram of Bishop calculation of roadbed with water erosion groove under vehicle loading.

Based on field investigation and relevant literature review, it can be found that the mechanism of water-damaged settlement failure of soil roadbed along the river is similar to that of rainfall-induced landslide, and the sliding surface is usually arc-shaped. When the soil roadbed along the river is washed by flood, a water erosion groove is formed at the slope toe, and the upper soil still meets the principle of limit equilibrium in a stable state. Therefore, based on the circular sliding mechanism, this study used Bishop method to analyze the stability of soil roadbed washout along the river. Bishop^13^ assumed that: (1) The acting force of soil between strips is only normal force, and there is no tangential force; (2) The sliding soil conforms to the overall moment balance condition; (3) Each block conforms to the polygonal closing condition of force; (4) The anti-sliding safety factor of the bottom sliding surface of roadbed soil strip is the same, which is equal to the average safety factor of the whole roadbed sliding surface. The soil slope per unit length is calculated as a plane problem. Set the landslide surface of roadbed as arc AC, the center of the circle as O and the radius as R. The landslide ABC is divided into several soil strips, and the stress of which is analyzed, as shown in Fig. 5. The stress on the ith soil strip is as below:

1. Weight of soil strip, Gi = γb_i_h_i_; Where, b_i_ and h_i_ are the width and average height of soil strip i; When there is a water erosion groove, Gi = γb_r_h_r_; Where, b_r_ and h_r_ are the residue width and residue height of soil strip i.
2. Shear resistance T_fi_, effective normal reaction N_i_' and pore water pressure u_i_l_i_ acting on the bottom surface of soil strip i, where, u_i_ and l_i_ are pore water pressure at the midpoint of the bottom surface of soil strip i and the length of sliding arc respectively; The Angle between the center line and the vertical of the soil strip i is α_i_.
3. Normal force E_i_ and E_i+1_ and tangential force X_i_ and X_i+1_ acting on both sides of soil strip i, ΔX_i_ = (X_i+1_ − X_i_).
4. Vehicle weight p_i_ and pavement slab weight q_i_ acting on soil strip i; Moreover, the action points of G_i_, T_fi_, N_i_′ and u_i_l_i_ are all at the midpoint of the bottom surface of the soil strip i.

For the vertical direction of soil strip i, according to the balance of force, the following formula can be obtained:

12 $$ G_{i} + \Delta X_{i} - T_{fi} \sin \alpha_{i} - N_{i}^{\prime } \cos \alpha_{i} - u_{i} l_{i} \cos \alpha_{i} + p_{i} + q_{i} = 0 $$

When the soil slope has not collapsed and remains stable, the shear strength T_fi_ on the sliding surface of soil strip i is expressed by effective stress, as below:

13 $$ T_{fi} = \frac{{\tau_{fi} l_{i} }}{K} = \frac{{{\text{c}^{\prime}}l_{i} }}{K} + N_{i}^{\prime } \frac{{\tan \varphi^{\prime}}}{K} $$

where, c′—Effective cohesion of soil, φ′—Effective internal friction angle of soil, K—Safety factor.

Substitute (12), and N_i_′ is

14 $$ N_{i}^{\prime } = \frac{1}{{m_{{a_{i} }} }}\left( {G_{i} + \Delta X_{i} - u_{i} b_{i} + p_{i} + q_{i} - \frac{{{\text{c}^{\prime}}l_{i} }}{K}\sin \alpha_{i} } \right) $$

where

15 $$ m_{{a_{i} }} = \cos \alpha_{i} \left( {1 + \frac{{\tan \varphi^{\prime} * \tan \alpha_{i} }}{k}} \right) $$

The moment balance analysis of the whole sliding soil body to the center O of the circle is carried out. Since the side wall action moments between adjacent soil strips cancel each other, and the action lines of N_i_' and u_i_l_i_ of each soil strip i pass through the center O of the circle, the following formula can be obtained:

16 $$ \sum {\left( {G_{i} + p_{i} + q_{i} } \right)} x_{i} - \sum {T_{fi} R = 0} $$

Substitute formulas (14) and (16) into formula (13), and x_i_ = Rsinα, b = b_i_ = l_i_cosα_i_. According to Bishop method, the safety factor of roadbed stability under the action of pavement slab and vehicle is as below:

17 $$ K = \frac{{\sum {\frac{1}{{m_{{a_{i} }} }}(c^{\prime}b + \left( {G_{i} + \Delta X_{i} - u_{i} b + p_{i} + q_{i} } \right)\tan \varphi^{\prime})} }}{{\sum {\left( {G_{i} + p_{i} + q_{i} } \right)} \sin \alpha_{i} }} $$

ΔX_i_ is estimated to determine K through the successive approximation method. The trial value of X_i_ and E_i_ should meet the equilibrium conditions of each soil strip. ∑ΔX_i_ and ∑ΔE_i_ of the whole sliding soil strip should be equal to zero. Bishop proved that the error is only 1% when ΔX_i_ = 0, and formula (17) can be simplified as follows:

18 $$ K = \frac{{\sum {\frac{1}{{m_{{a_{i} }} }}\left( {c^{\prime}b + \left( {G_{i} - u_{i} b + p_{i} + q_{i} } \right)\tan \varphi^{\prime}} \right)} }}{{\sum {\left( {G_{i} + p_{i} + q_{i} } \right)} \sin \alpha_{i} }} $$

### Stability analysis of roadbed under flood scouring

#### Stability analysis of roadbed under straight flood scouring

**Figure 6:**
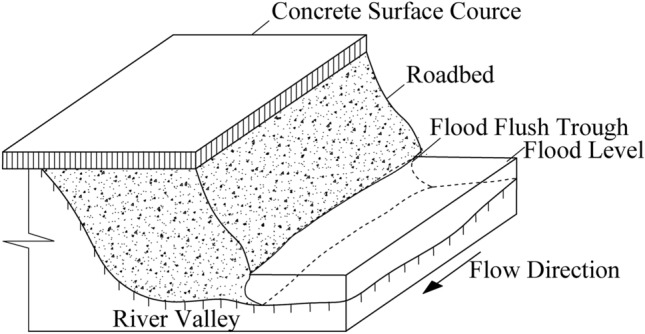
Diagram of straight flood scouring roadbed along the river.

Considering that the scouring force is parallel to the longitudinal section of the roadbed when the flood scours straightly along the river, as shown in Fig. 6, the moment to the center O of the circle is 0. Therefore, formula (18) can be used to calculate for calculating the stability safety factor of soil roadbed along the river in the straight section.

#### Stability analysis of roadbed under flood bend

**Figure 7:**
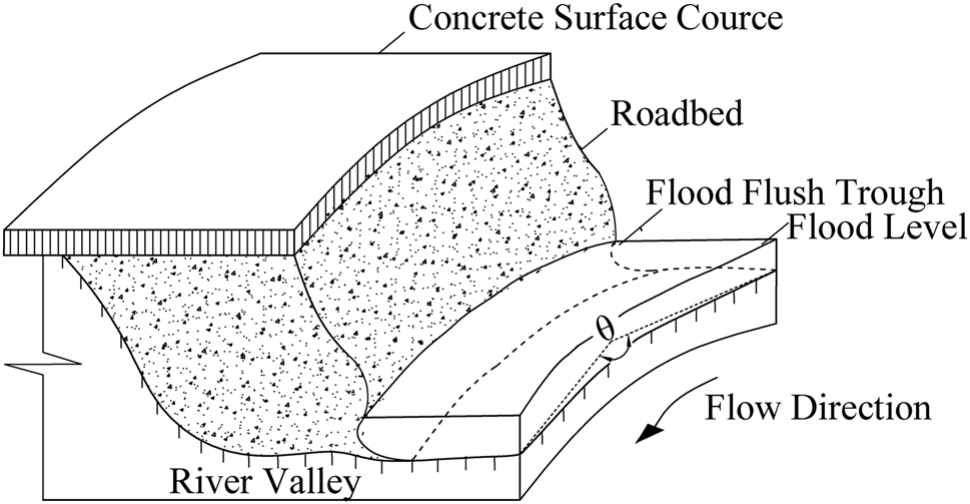
Diagram of bend flood scouring roadbed along the river.

**Figure 8:**
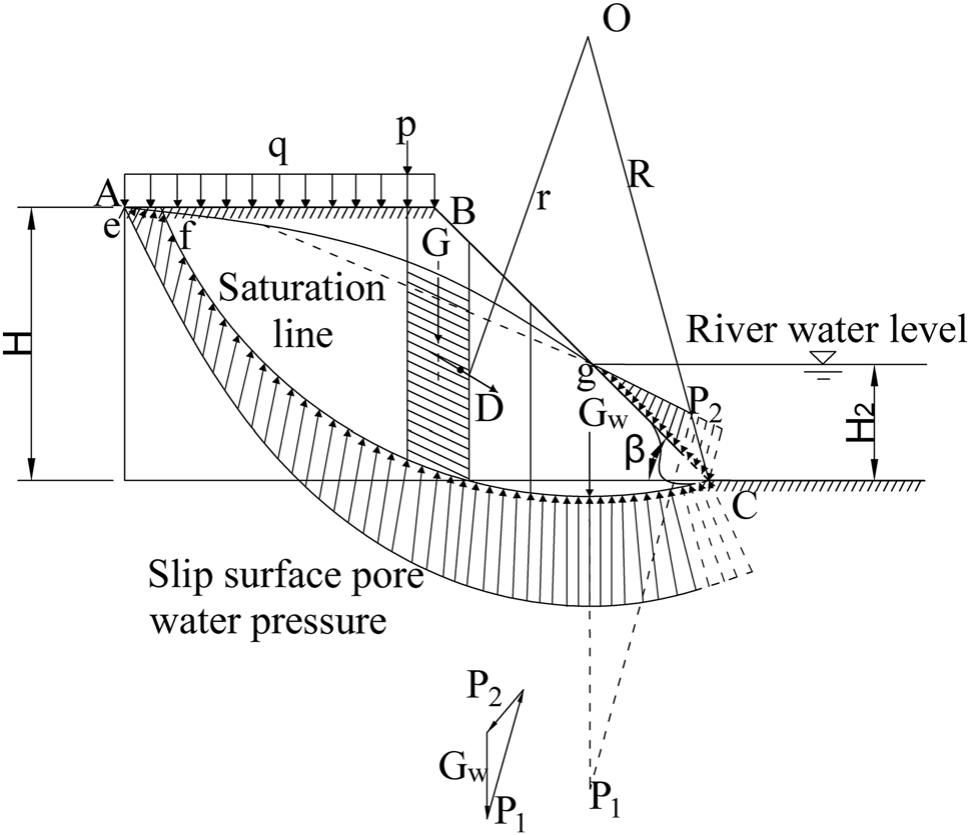
Calculation of roadbed stability during water seepage.

When the flood bends scour the soil roadbed along the river, the water flow acts perpendicularly to the longitudinal section of the soil roadbed, as shown in Fig. 7. The flood scouring force is simplified as concentrated force F. When it acts on the center line of the flood level, the moment to the center O of the circle is $M = F\left( {R - \frac{{H_{2} }}{{2\sin \beta \cos \beta_{1} }}} \right)\cos \beta_{1}$. Where β is the toe of the slope and β_1_ is ∠OCB, as shown in Fig. 8. In this study, in order to calculate the most dangerous state, let F act on the most dangerous position, that is, the toe of soil roadbed slope, and the moment of the center O of the circle can be simplified as M = FR.

According to the momentum theorem Ft = 2mv_⊥_, where v_⊥_ is the velocity of water flow perpendicular to the river bank in the concave part of the river, v_⊥_ = vcos(θ/2), where v is the river flow velocity, θ is the angle of the river bend, m = ρv = ρQt, and Q is the flow of the river bend, F = 2ρQv_⊥_ is obtained, which is substituted into (18), and the stability safety factor at the bend along the concave part of the river road is obtained as follows:

19 $$ K = \frac{{\sum {\frac{1}{{m_{{a_{i} }} }}(c^{\prime}b + \left( {G_{i} - u_{i} b + p_{i} + q_{i} } \right)\tan \varphi^{\prime}) + 2\rho Qv_{ \bot } R} }}{{\sum {\left( {G_{i} + p_{i} + q_{i} } \right)} \sin \alpha_{i} }} $$

When the soil slope is partially flooded. The hydrostatic pressure P_1_ acting on the sliding surface below the water level line, the water pressure P_2_ on the slope surface, the gravity of pore water and the reaction force G_w_ of soil particle buoyancy are balanced in the hydrostatic state. The action line of P_1_ passes through the center O of the circle. According to the moment balance, the moment of P_2_ to the center O of the circle and the moment of G_w_ to the center O of the circle cancel each other, as shown in Fig. 8. Therefore, under the condition of hydrostatic state, the influence of water pressure on roadbed surface on the sliding soil can be calculated according to the buoyancy of the sliding soil below the still water surface, that is, the weight of the underwater soil strip is calculated according to the effective weight. The calculation formula of the stability safety factor is the same as formula (19).

When it rains continuously, the mountain surface water flows downwards and seeps into the river through the roadbed. The water level in the slope is higher than that outside the slope, and the water in the slope generates seepage force outward, pointing to the slope surface, as shown in Fig. 8. Assuming that the saturation line is e–f–g, the area of the part (f–g–c) of the sliding soil under the saturation line is A_w_, and the total seepage force D on the soil is as below:

20 $$ D = JA_{w} = \gamma_{w} iA_{w} $$

where, J—Seepage force on unit volume of soil (KN/m^3^); i—The average value of hydraulic gradient in the area A_w_ under the saturation line, i is assumed to be equal to the gradient of the connection line of the saturation line f-g. γ_w_—Saturated water severity.

The action line of the seepage resultant force D is located at the centroid of area f-g-c, assuming that the action direction is parallel to f-g, and the arm of force of D to the center O of the circle is recorded as r. Therefore, considering the seepage force, formula (19) is substituted and the formula for analyzing the stability safety factor of roadbed along the river by Bishop method is as follows:

21 $$ K = \frac{{\sum {\frac{1}{{m_{{a_{i} }} }}(c^{\prime}b + \left( {G_{i} - u_{i} b + p_{i} + q_{i} } \right)\tan \varphi^{\prime}) + 2\rho Qv_{ \bot } R} }}{{\sum {\left( {G_{i} + p_{i} + q_{i} } \right)} \sin \alpha_{i} + \frac{r}{R}D}} $$

**Figure 9:**
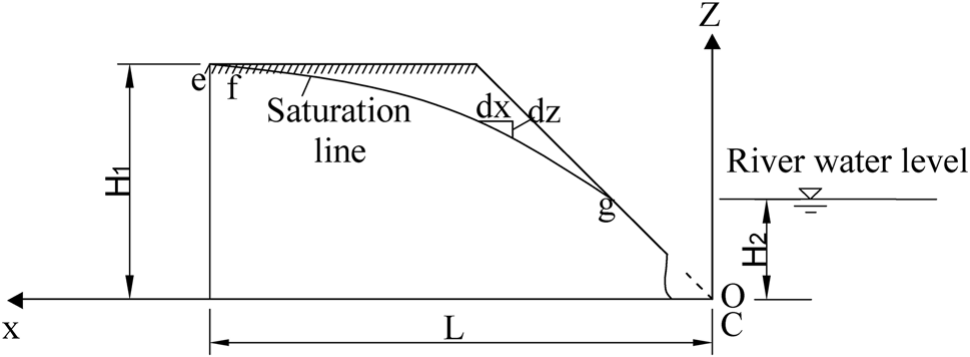
Seepage calculation of impervious roadbed soil.

Homogeneous, laminar and stable gradual flow are assumed according to the hydraulics^15–17^, as shown in Fig. 9.

The upper water level is H_1_, which can be approximated as the height H of roadbed along the river in this study. The height of lower water level is the height H_2_ of river water level, and the horizontal projection length of roadbed bottom is L. According to Darcy's law, the average velocity of roadbed across the river is as below:

22 $$ \overline{v} = - k\frac{dz}{{dx}} $$

The seepage flow is q_s_:

23 $$ q_{s} = \overline{v}z = - kz\frac{dz}{{dx}} $$

The variables are separated from the above formula, and the upstream surface ($x = 0,{\text{z}} = H_{1}$) H is integrated to the downstream surface ($x = L,z = H_{2}$):

24 $$ H_{1}^{2} - H_{2}^{2} = \frac{{2q_{s} }}{k}L $$

25 $$ q_{s} = \frac{{k\left( {H_{1}^{2} - H_{2}^{2} } \right)}}{2L} $$

If the integral limit is changed to x = 0 ~ x, z = H_1_ ~ z, formula (25) becomes the equation of the saturation line.

26 $$ q_{s} = \frac{{k\left( {H_{1}^{2} - z^{2} } \right)}}{2x} $$

From formula (26),

27 $$ z = \sqrt {H_{1}^{2} - \frac{{2q_{s} }}{k}x} $$

Substitute (25) into (27)

28 $$ z = \sqrt {H_{1}^{2} - \frac{{H_{1}^{2} - H_{2}^{2} }}{Lk}x} $$

According to the saturation line formula (28), Aw can be obtained.

29 $$ \begin{aligned} A_{w} & = \int_{0}^{L} {\sqrt {H_{1}^{2} - \frac{{H_{1}^{2} - H_{2}^{2} }}{L}x} } dx \\ & = \frac{{2L\left( {H_{1}^{3} - H_{2}^{3} } \right)}}{{3\left( {H_{1}^{2} - H_{2}^{2} } \right)}} \\ & = \frac{{2L\left( {H^{3} - H_{2}^{3} } \right)}}{{3\left( {H^{2} - H_{2}^{2} } \right)}} \\ \end{aligned} $$

Equations (28) and (29) are substituted into (21), the following formula can be obtained:

30 $$ K = \frac{{\sum {\frac{1}{{m_{{a_{i} }} }}(c^{\prime}b + \left( {G_{i} - u_{i} b + p_{i} + q_{i} } \right)\tan \varphi^{\prime}) + 2\rho Qv_{ \bot } R} }}{{\sum {\left( {G_{i} + p_{i} + q_{i} } \right)} \sin \alpha_{i} + \frac{{2r\gamma_{w} iL\left( {H^{3} - H_{2}^{3} } \right)}}{{3R\left( {H^{2} - H_{2}^{2} } \right)}}}} $$

### Prediction and analysis of roadbed erosion by river

In order to calculate the stability safety factor of the residue roadbed soil of the highway along the river in water erosion, the self-weight of soil strips is recorded as G_ir_ = γb_ir_h_ir_, where, b_ir_ and h_ir_ are the width and height of soil strip i of the residue roadbed soil after river water erosion, respectively, and they are substituted into Formula (30).

31 $$ K = \frac{{\sum {\frac{1}{{m_{{a_{i} }} }}(c^{\prime}b_{ir} + \left( {\gamma b_{ir} h_{ir} - u_{i} b_{ir} + p_{i} + q_{i} } \right)\tan \varphi^{\prime}) + 2\rho Qv_{ \bot } R} }}{{\sum {\left( {\gamma b_{ir} h_{ir} + p_{i} + q_{i} } \right)} \sin \alpha_{i} + \frac{{2r\gamma_{w} iL\left( {H^{3} - H_{2}^{3} } \right)}}{{3R\left( {H^{2} - H_{2}^{2} } \right)}}}} $$

**Figure 10:**
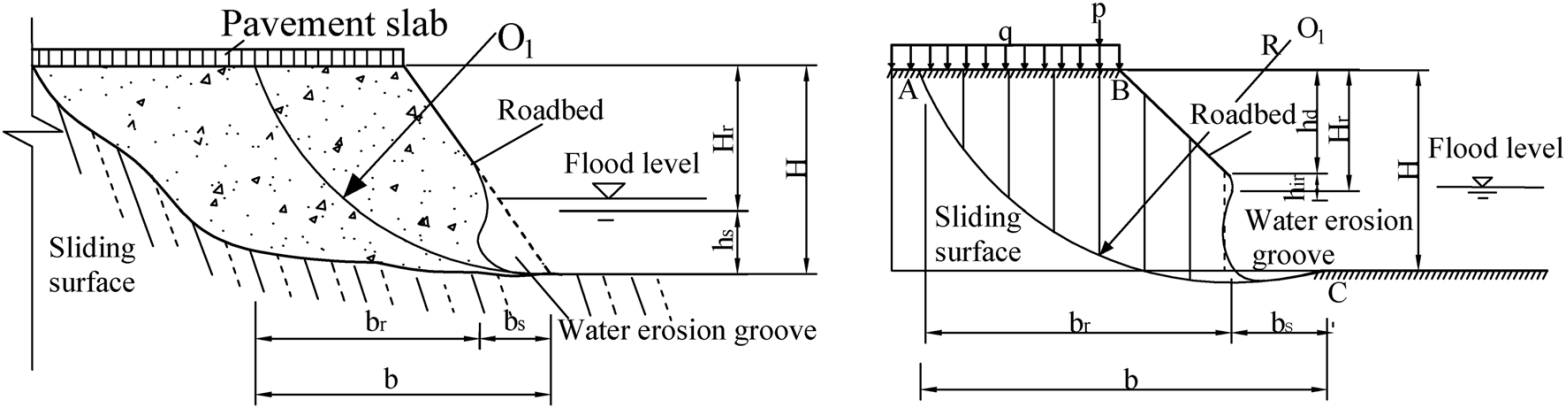
Schematic diagram of flood water erosion parameters.

In the state of smooth revetment, as shown in Fig. 10, the roadbed along the river is partially scoured by flood. Based on the scour depth calculation formula of smooth revetment in *Code for River Regulation Design* (GB50707-2011)^14^, the scour depth is as below:

32 $$ b_{s} = H_{2} *\left[ {\left( {\frac{{v_{ \bot } }}{{u_{c} }}} \right)^{n} - 1} \right] $$

where, b_s_-Local scour depth from roadbed bottom (m); H_2_-Water depth at scouring position (m); v_⊥_-Average vertical velocity near the shore (m/s); u_c_-Sediment incipient velocity (m/s).

According to the stable sliding surface obtained by the Bishop method, combined with formula (32), the residue stable roadbed width is obtained as follows:

33 $$ b_{r} = b - b_{s} = b - H_{2} *\left[ {\left( {\frac{{v_{ \bot } }}{{u_{c} }}} \right)^{n} - 1} \right] $$

Based on formula (33), the calculation formula of h_r_ corresponding to the critical soil strip for b_r_ is as follows:

34 $$ h_{r} = \frac{{\sum {\frac{1}{{m_{{a_{i} }} }}\left( {c^{\prime}b_{ir} + \left( {p_{i} + q_{i} - u_{i} b_{ir} } \right)\tan \varphi^{\prime}} \right) + 2\rho Qv_{ \bot } R - \frac{{2Kr\gamma_{w} iL\left( {H^{3} - H_{2}^{3} } \right)}}{{3R\left( {H^{2} - H_{2}^{2} } \right)}} - K\sum {\left( {p_{i} + q_{i} } \right)} \sin \alpha_{i} } }}{{K\sum {\gamma b_{ir} } \sin \alpha_{i} - \sum {\frac{{\gamma b_{ir} \tan \varphi^{\prime}}}{{m_{{a_{i} }} }}} }} $$

According to the vertical distance h_d_ between the roadbed drawn by the Bishop method and the vertex of the center line of unstable soil strip i, it is concluded that the height of the residual stable roadbed soil after river scouring and water erosion is H_r_.

35 $$ \begin{gathered} H_{r} = h_{d} + h_{r} \\ = h_{d} + \frac{{\sum {\frac{1}{{m_{{a_{i} }} }}(c^{\prime}b_{ir} + \left( {p_{i} + q_{i} - u_{i} b_{ir} } \right)\tan \varphi^{\prime}) + 2\rho Qv_{ \bot } R - \frac{{2Kr\gamma_{w} iL\left( {H^{3} - H_{2}^{3} } \right)}}{{3R\left( {H^{2} - H_{2}^{2} } \right)}} - K\sum {\left( {p_{i} + q_{i} } \right)} \sin \alpha_{i} } }}{{K\sum {\gamma b_{ir} } \sin \alpha_{i} - \sum {\frac{{\gamma b_{ir} \tan \varphi^{\prime}}}{{m_{{a_{i} }} }}} }} \\ \end{gathered} $$

The height of the roadbed bottom eroded by river water is as follows:

36 $$ \begin{gathered} h_{s} = H - H_{r} \\ = H - h_{d} - \frac{{\sum {\frac{1}{{m_{{a_{i} }} }}(c^{\prime}b_{ir} + \left( {p_{i} + q_{i} - u_{i} b_{ir} } \right)\tan \varphi^{\prime}) + 2\rho Qv_{ \bot } R - \frac{{2Kr\gamma_{w} iL\left( {H^{3} - H_{2}^{3} } \right)}}{{3R\left( {H^{2} - H_{2}^{2} } \right)}} - K\sum {\left( {p_{i} + q_{i} } \right)} \sin \alpha_{i} } }}{{K\sum {\gamma b_{ir} } \sin \alpha_{i} - \sum {\frac{{\gamma b_{ir} \tan \varphi^{\prime}}}{{m_{{a_{i} }} }}} }} \\ \end{gathered} $$

In order to ensure the safety of highway operation and protect the stability of pavement slab and roadbed, the limit value of passing vehicles should be obtained^18^. Therefore, according to formula (36), the vehicle load P_l_ is as below:

37 $$ P_{l} = \frac{{\sum {\frac{1}{{m_{{a_{i} }} }}(c^{\prime}b + \left( {\gamma b_{ir} h_{ir} + q_{i} - u_{i} b} \right)\tan \varphi^{\prime}) + 2\rho Qv_{ \bot } R - \frac{{2Krr_{w} iL\left( {H^{3} - H_{2}^{3} } \right)}}{{3R\left( {H^{2} - H_{2}^{2} } \right)}} - K\sum {\left( {\gamma b_{ir} h_{ir} + q_{i} } \right)} \sin \alpha_{i} } }}{{K\sin \alpha_{i} - \sum {\frac{{\tan \varphi^{\prime}}}{{m_{{a_{i} }} }}} }} $$

## Case analysis

The bend near K4 + 210 third-class highway in Shizhu County, Chongqing is taken as an example. This section is a semi-filled and semi-dug soil roadbed, with soil roadbed height H = 5 m and pavement width b = 4.0 m. The section along the river is a homogeneous soil-rock mixture slope. For the semi-filled and semi-dug roadbed, the medium-dense pebble soil, gravel soil, rounded gravel soil and breccia soil are used for filling, and the particle size of sand is less than 150 mm. The slope is a Class IV strong wind fossil slope, which is affected by groundwater^19,20^. The filling slope is 1:1. According to the Bishop's method, soil strips are divided into 15 strips, and the stability safety factor K of soil slope is calculated by Formulas (18–37). The changing height and width of scouring water erosion at the bottom of roadbed along the river and the load limit value of vehicles passing on the road are calculated and predicted. Physical and mechanical indexes of roadbed filler are shown in Table 1.

**Table 1:** Physical and mechanical indexes of roadbed filler.

Roadbed soil quality	Saturated unit weight/KN/m^3^	Natural density/KN/m^3^	Effective cohesion c′/KPa	Internal friction angle φ′/°	Self-weight force of pavement slab q/kN	Vehicle force p/KN
Material	20.50	20.00	15.60	22	17.8	200
Pore water pressure at the midpoint of soil strip bottom surface u_i_	Flood density T/m^3^	Flood bend discharge Q, m3/s	R radius, m	Arm of force of D to the center O r,m	Friction coefficient of base	Friction coefficient of base
14	1.014	34	7.4668	5.9775	0.25	0.25

### Sensitivity analysis of water depth, flow velocity, river bending angle and stability safety factor

#### Sensitivity analysis of water depth, river bending angle and stability safety factor

**Figure 11:**
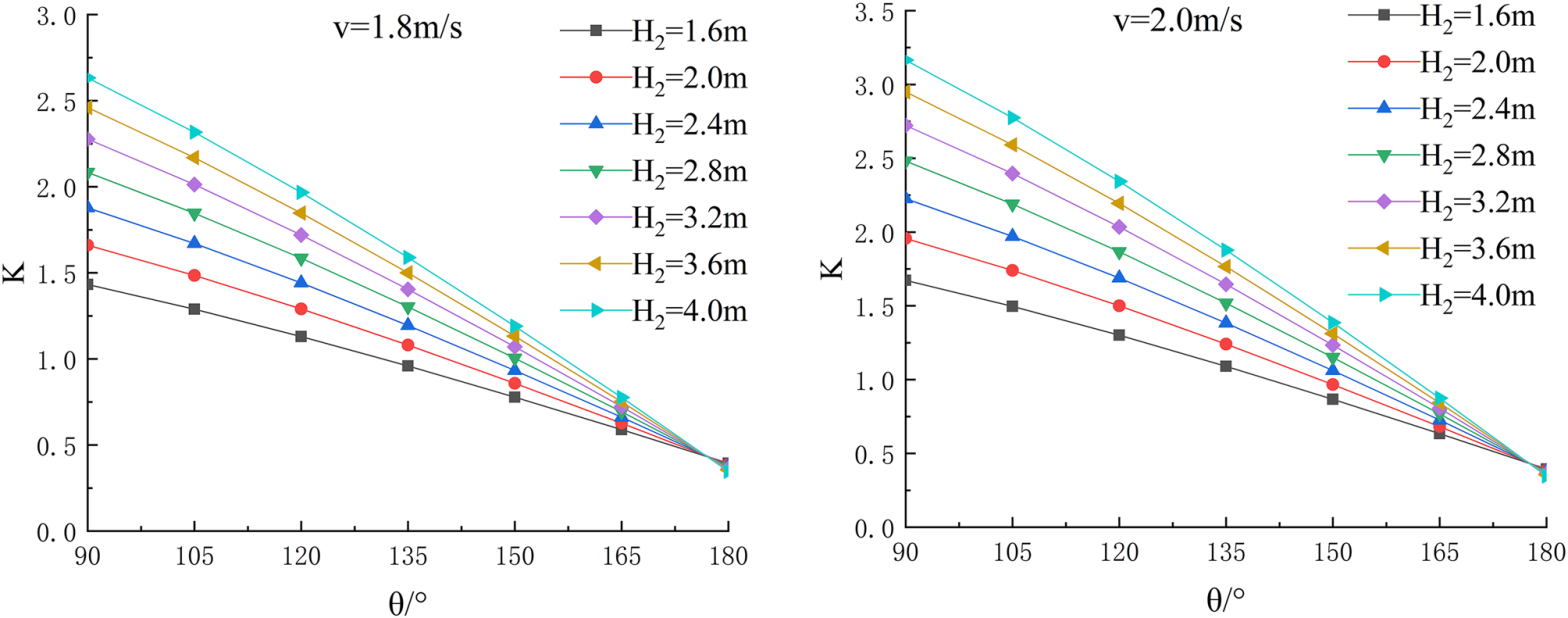
Sensitivity analysis of different H_2_ and θ to K when V = 1.8 m/s, 2.0 m/s.

**Figure 12:**
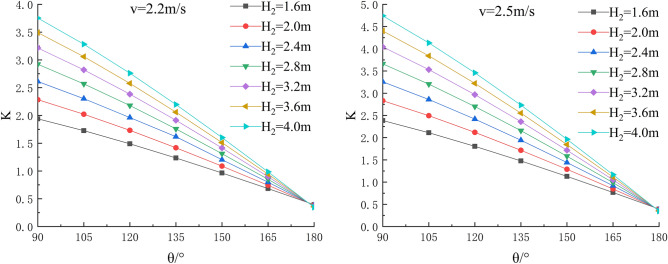
Sensitivity analysis of different H_2_ and θ to K when V = 2.2 m/s, 2.5 m/s.

**Figure 13:**
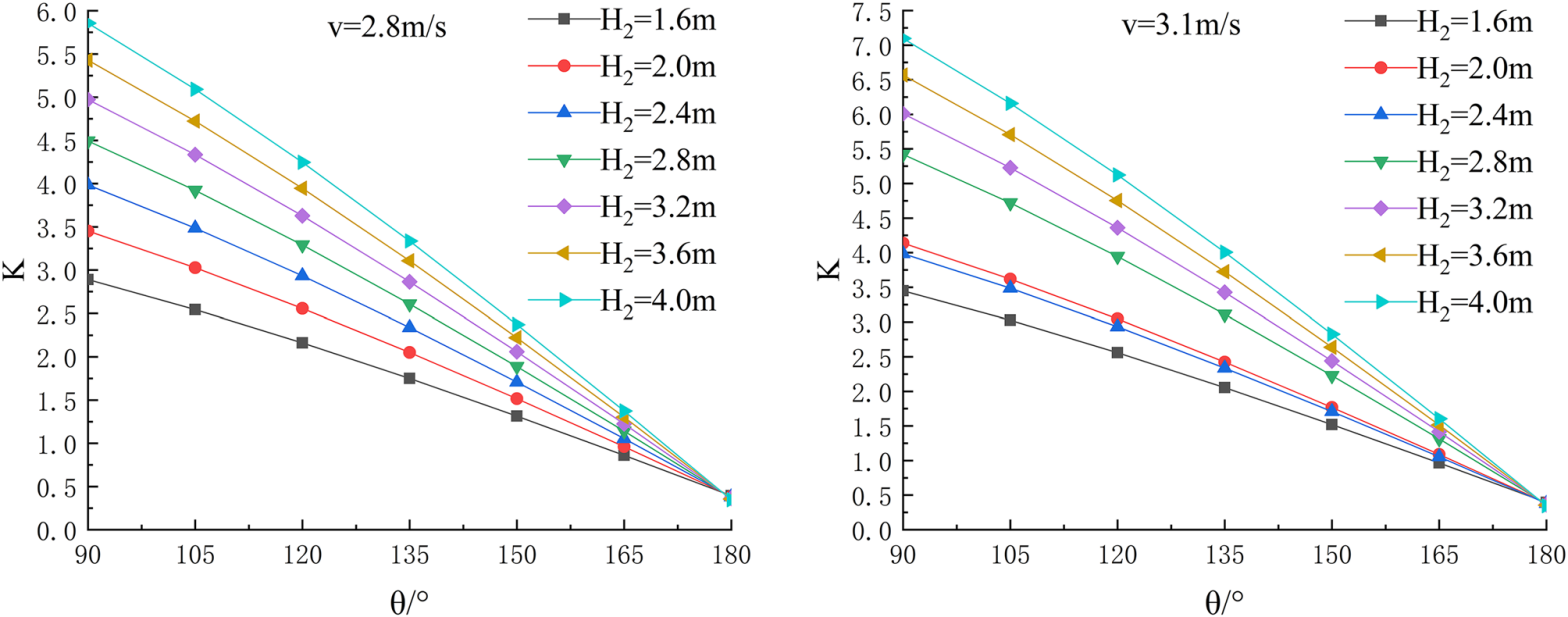
Sensitivity analysis of different H_2_ and θ to K when V = 2.8 m/s, 3.1 m/s.

In the depth of the water, river bending angle and the stability safety factor of sensitivity analysis, working condition of parameter selection are as follows: Roadbed slope along the river, the slope toe slope ratio is 1:1, the flood depth is H_2_ = 1.6 m, 2.0 m, 2.4 m, 2.8 m, 3.6 m and 4.0 m; respectively, and the water flow velocity is 1.8 m/s, 2.0 m/s, 2.2 m/s, 2.5 m/s, 2.8 m/s and 3.1 m/s. The bending angle at the concave bank of the smooth revetment river is θ = 90°,105°,120°,135°,150°,165°,180°; as shown in Figs. 11, 12, 13.

#### Sensitivity analysis of flow velocity, river bending angle and stability safety factor

**Figure 14:**
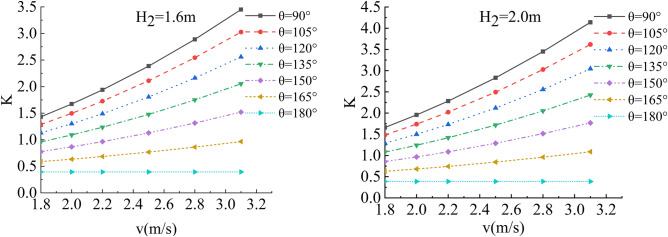
Sensitivity analysis of different v and θ to K when H_2_ = 1.6 m/s, 2.0 m/s.

**Figure 15:**
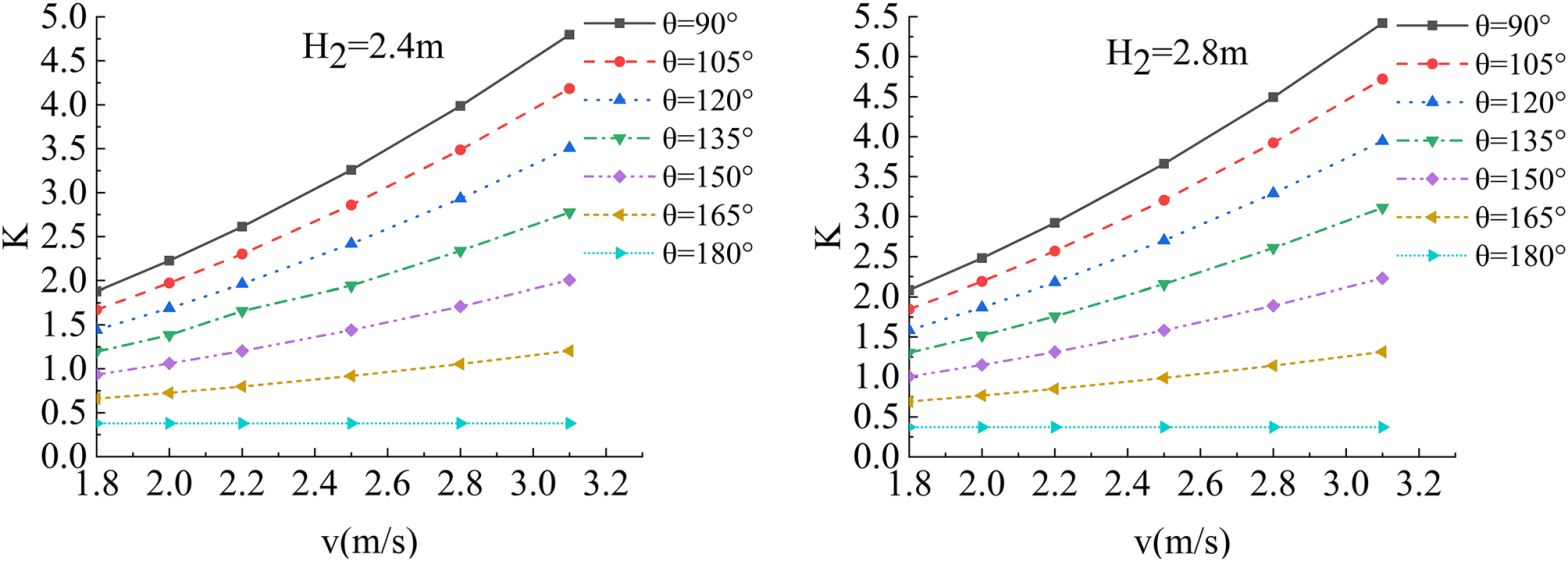
Sensitivity analysis of different v and θ to K when H_2_ = 2.4 m/s, 2.8 m/s.

**Figure 16:**
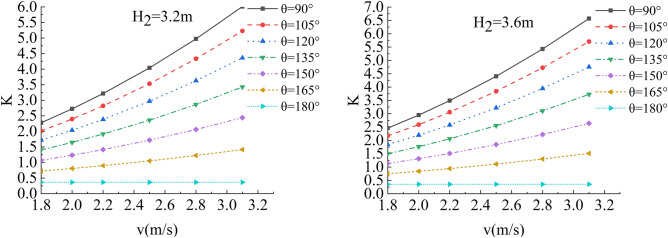
Sensitivity analysis of different v and θ to K when H_2_ = 3.2 m/s,3.6 m/s.

**Figure 17:**
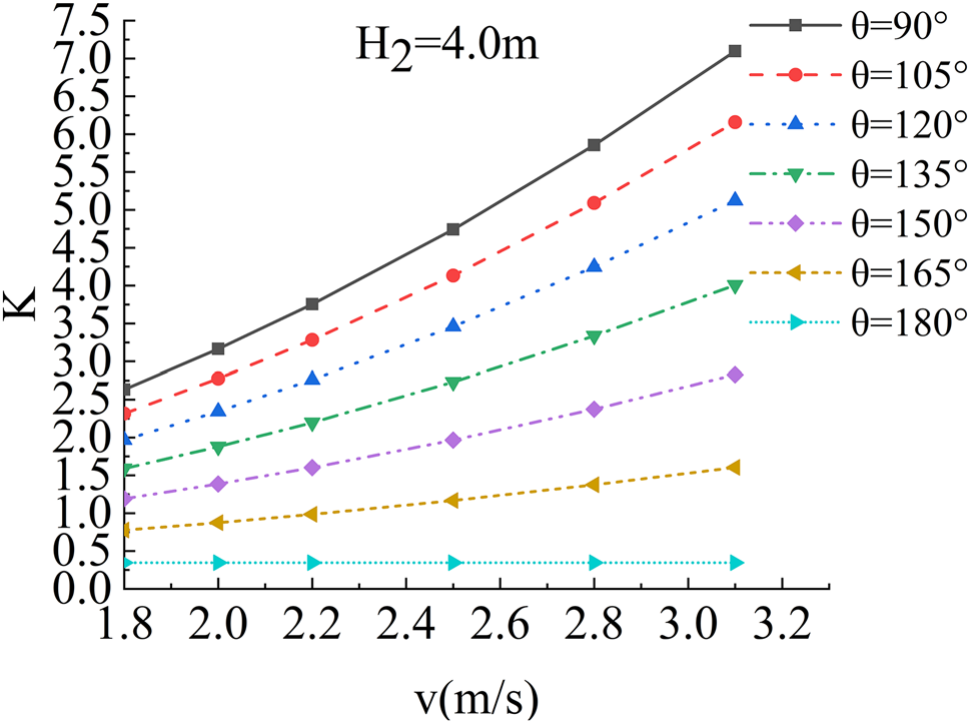
Sensitivity analysis of different v and θ to K when H_2_ = 4.0 m/s.

In the flow velocity, river bending angle and stability safety factor of sensitivity analysis, working condition of parameter selection are as follows: Roadbed slope along the river, the slope toe slope ratio is 1:1, the flood depth is 1.6 m, 2 m, 2.4 m, 2.8 m, 3.2 m, 3.6 m and 4.0 m respectively, and the water flow velocity is 1.8 m/s, 2.0 m/s, 2.2 m/s, 2.5 m/s, 2.8 m/s and 3.1 m/s. The bending angle at the concave bank of the smooth revetment river is θ = 90°,105°,120°,135°,150°,165°,180°; as shown in Figs. 14, 15, 16 and 17.

Relevant parameters of roadbed and river are selected for the above working conditions. After the design and construction of roadbed is completed, although the design K value has been determined, the sensitivity analysis of the demand value of stability safety factor for the above common working conditions of roadbed along the river under flood and water erosion can be taken as a reference to guide the safety and protection of roadbed according to the following analysis conclusions:

1. When the flood level H_2_ changes from 1.6 m to 4.0 m, the demand value of stability safety factor K of soil roadbed increases with the increase of the water depth;
2. When the river bend angle θ changes from 90° to 180°, the demand value of stable safety factor K decreases with the increase of the river bend angle;
3. When the water depth and velocity are fixed, the required value of stability safety factor K decreases with the increase of the river bending angle.
4. When the water depth is fixed, the required value of stability safety factor K increases with the increase of river velocity, and the increase rate decreases with the increase of the river bending angle. Particularly, the required value of stability safety factor K is constant for straight river section.
5. When the flow rate is fixed and the river bend angle is the same, the demand value of stability safety factor K increases with the increase of the water depth;
6. When the flow rate is fixed and the bend angle of the river becomes larger, the required value of the stability safety factor K decreases with the increase of the water depth.
7. By comparing the required value K with the original design value, it can be judged whether the soil roadbed along the river exceeds the original design stable state under the corresponding working conditions. When the required value of K is smaller than the original design value, the soil roadbed along the river is safe under the corresponding working conditions. When the required value of K is close to the original design value, it is in a critical state, so it is suggested to take corresponding reinforcement measures; As the required value of K is larger than or equal to the original design value, the soil roadbed along the river is likely to be damaged, and corresponding preventive measures should be taken. Therefore, by comparing the required value of K with the original design value, it can be timely and accurately judged whether the soil roadbed along the river is in a safe state under the corresponding working conditions to take corresponding reinforcement and damage prevention measures, so that pre-judgment and decision-making for disaster prevention and mitigation can be realized in advance.

### Sensitivity analysis of water depth, velocity, river bending angle and soil roadbed along the river b_r_ and h_r_

**Figure 18:**
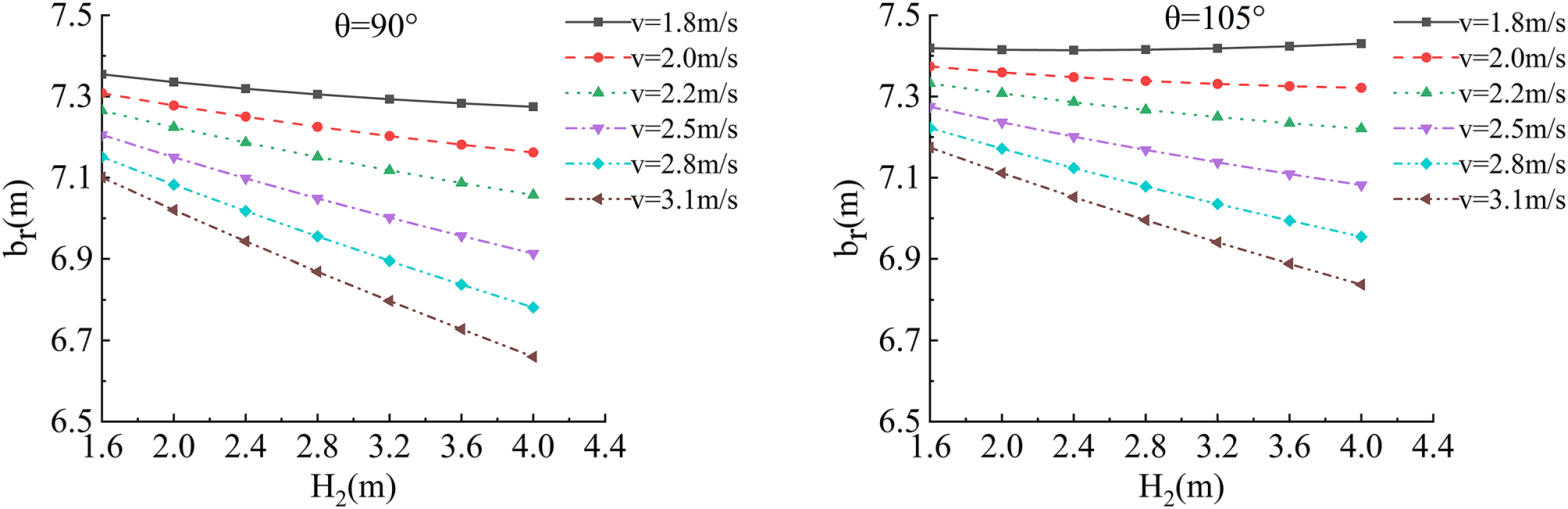
Sensitivity analysis of different v, H_2_ and b_r_ of the roadbed along the river when θ = 90°, 105°.

**Figure 19:**
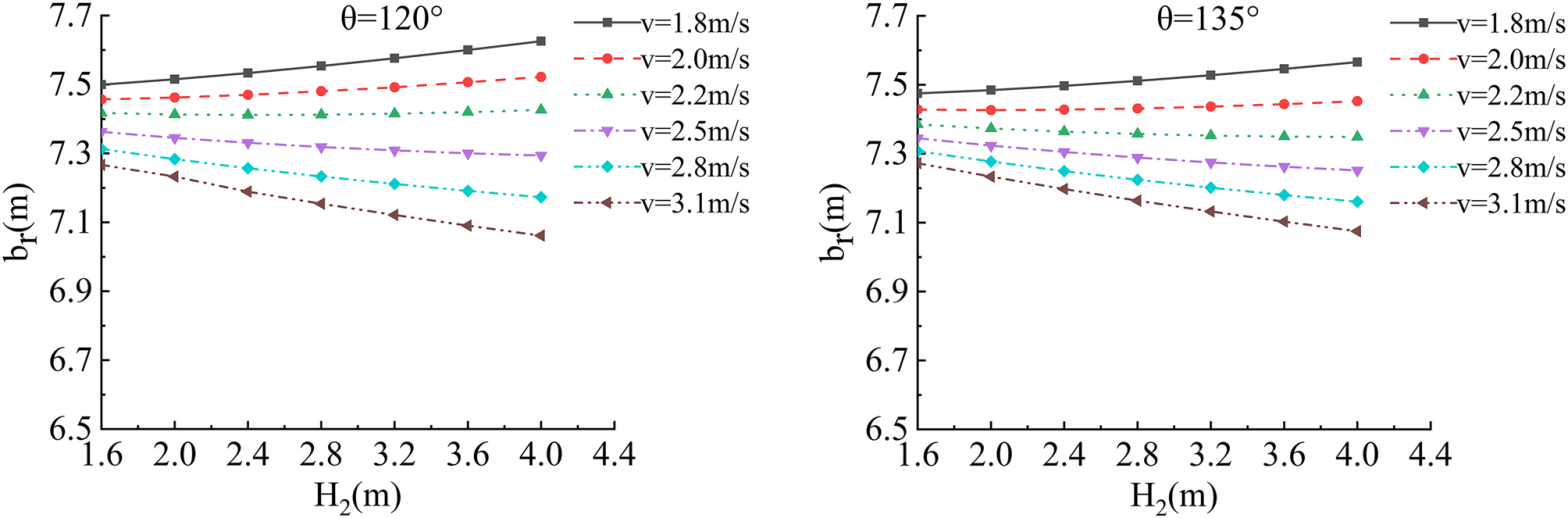
Sensitivity analysis of different v, H_2_ and b_r_ of the roadbed along the river when θ = 120°, 135°.

**Figure 20:**
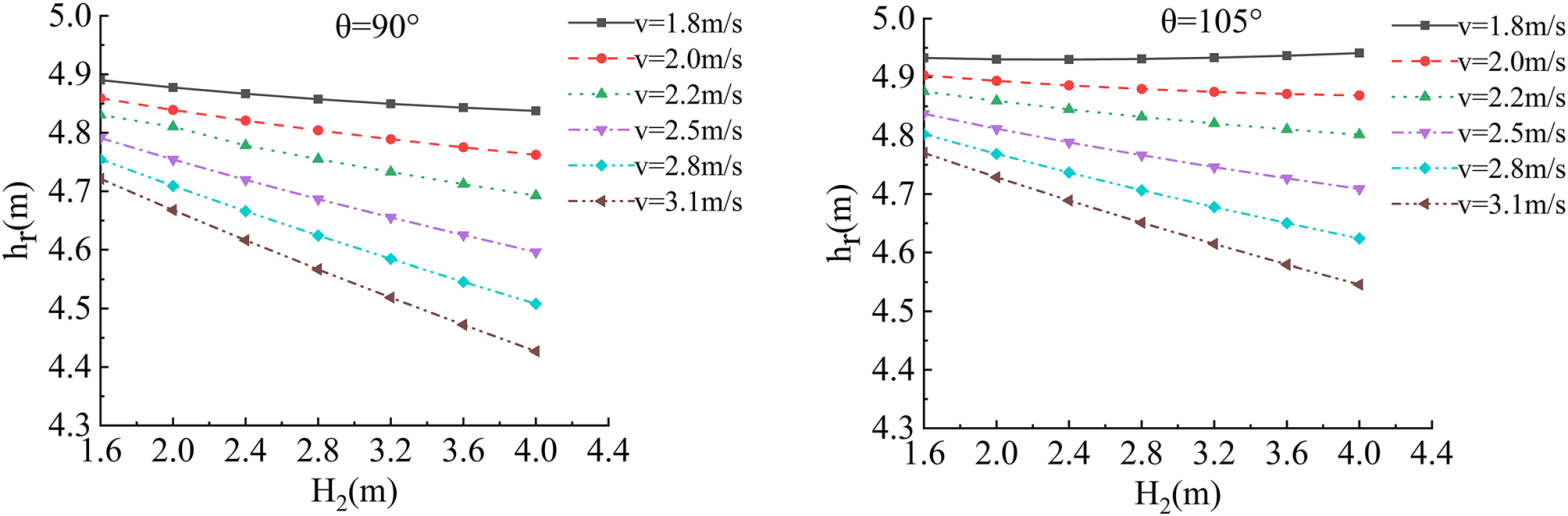
Sensitivity analysis of different v, H_2_ and h_r_ of the roadbed along the river when θ = 90°, 105°.

**Figure 21:**
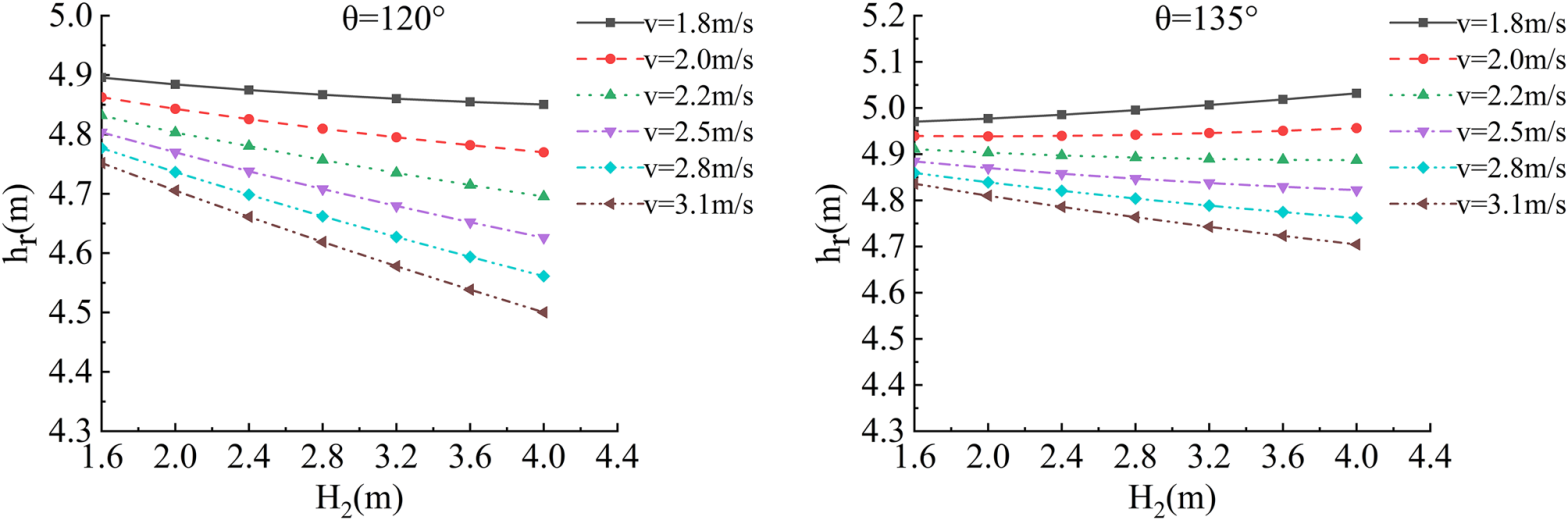
Sensitivity analysis of different v, H_2_ and h_r_ of the roadbed along the river when θ = 120°, 135°.

In the depth of the water, flow velocity, river bending angle and soil roadbed along the river b_r_ and h_r_ of sensitivity analysis, working condition of parameter selection are as follows: The slope toe gradient of roadbed along the river is 45°, 1:1, smooth revetment, the flood depth is H_2_ = 1.6 m, 2.0 m, 2.4 m, 2.8 m, 3.6 m and 4.0 m; the water flow velocity is v = 1.8 m/s, 2.0 m/s, 2.2 m/s, 2.5 m/s, 2.8 m/s and 3.1 m/s, and the typical river bending angle is θ = 90°,105°,120°,135° (Biswas et al.^15^), as shown in Figs. 18, 19, 20 and 21.

In this working condition, the common parameters of soil roadbed and river are selected. When the soil roadbed along the river at different river bending angles is scoured under different river flow rates, the width and height of residue roadbed soil change. Through the sensitivity analysis of flow rate, river bending angle and river water level on the width and height dimensions of residue soil roadbed along the river, the following conclusions can be drawn:

1. When θ ≤ 120°, the erosion damage of the slope and toe of soil roadbed along the river increases with the increase of water depth and velocity, and the corresponding values b_r_ and h_r_ decrease.
2. When θ = 90°, the slope and toe of soil roadbed along the river suffer from the strongest scouring destructive power, the corresponding b_r_ and h_r_ decrease the most rapidly, and their corresponding scouring change rate are also the largest. However, with the increase of bending angle, the change rate of the scouring decreases.
3. The river bending angle is the most obvious factor that affects the slope and toe of soil roadbed along the river. Particularly when the river bending angle is 90°, slope protection measures should be taken.
4. When the river bending angle is θ = 135°, it can be seen from the calculation results that v = 2.2 m/s is the scouring boundary value of soil roadbed slope and toe along the river. when v ≤ 2.2 m/s, b_r_ and h_r_ increase slightly with the increase of water depth which may be due to the slow flow speed and the increase of water depth, resulting in sediment deposition on soil roadbed slope and toe. When v > 2.2 m/s, the change law is consistent with that when θ ≤ 120°.
5. To sum up, the suspended pavement of soil roadbed along the river is mostly located in the concave bank, and the river bending angle is 90° ~ 135°.

## Numerical simulation analysis of roadbed along the river under flood scouring

**Figure 22:**
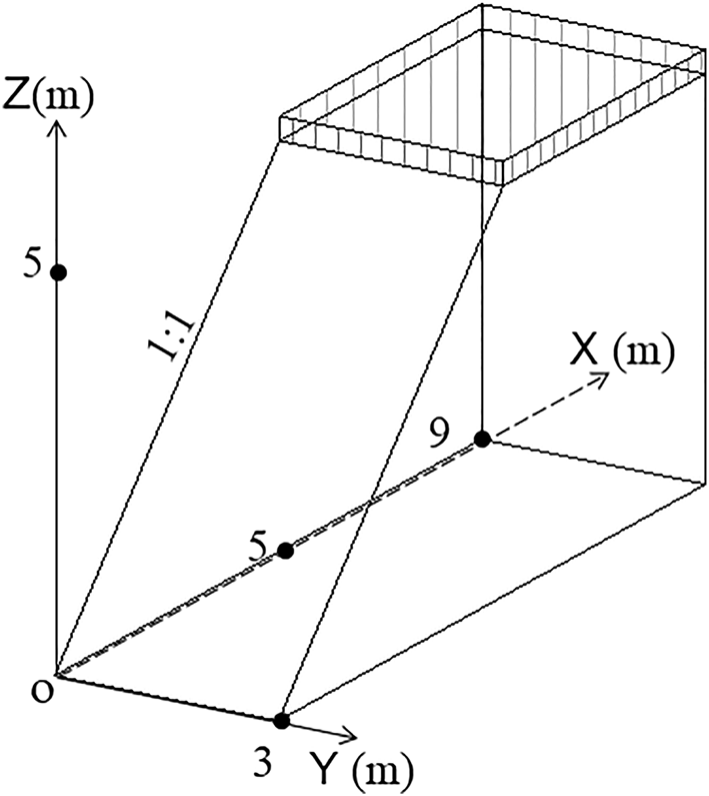
Diagram of numerical simulation model.

The model was established by FLAC3D: The size of the pavement slab and the soil roadbed is 5 m × 4.0 m × 0.3 m and 5 m × 4.0 m × 3 m respectively, and the slope is 1:1, as shown in Fig. 22. The material parameters of this model are listed in Table 2. The model adopts automatic grid division, with 131,327 units and 117,677 nodes. The scouring simulation of soil roadbed slope along the river was carried out in turn under the river water depth of 1.6 m, 2.0 m, 2.4 m, 2.8 m, 3.2 m, 3.6 m and 4.0 m, the water flow velocity of 2.2 m/s, and the river bend angle of 90°. The changing trend of displacement and stress of roadbed soil was verified when the road vehicle was driven by a single wheel with 100KN against the outside of roadbed slope and the pavement slab.

**Table 2:** Physical and mechanical indexes.

Material	Compressive Modulus of resilience/MPa	Density/kN/m^3^	Poisson’s ratio	Internal friction angle/°	Cohesion/KPa
C30	31,000	2500	0.15	-	-
Roadbed soil	35	2000	0.35	22	15.6

**Figure 23:**
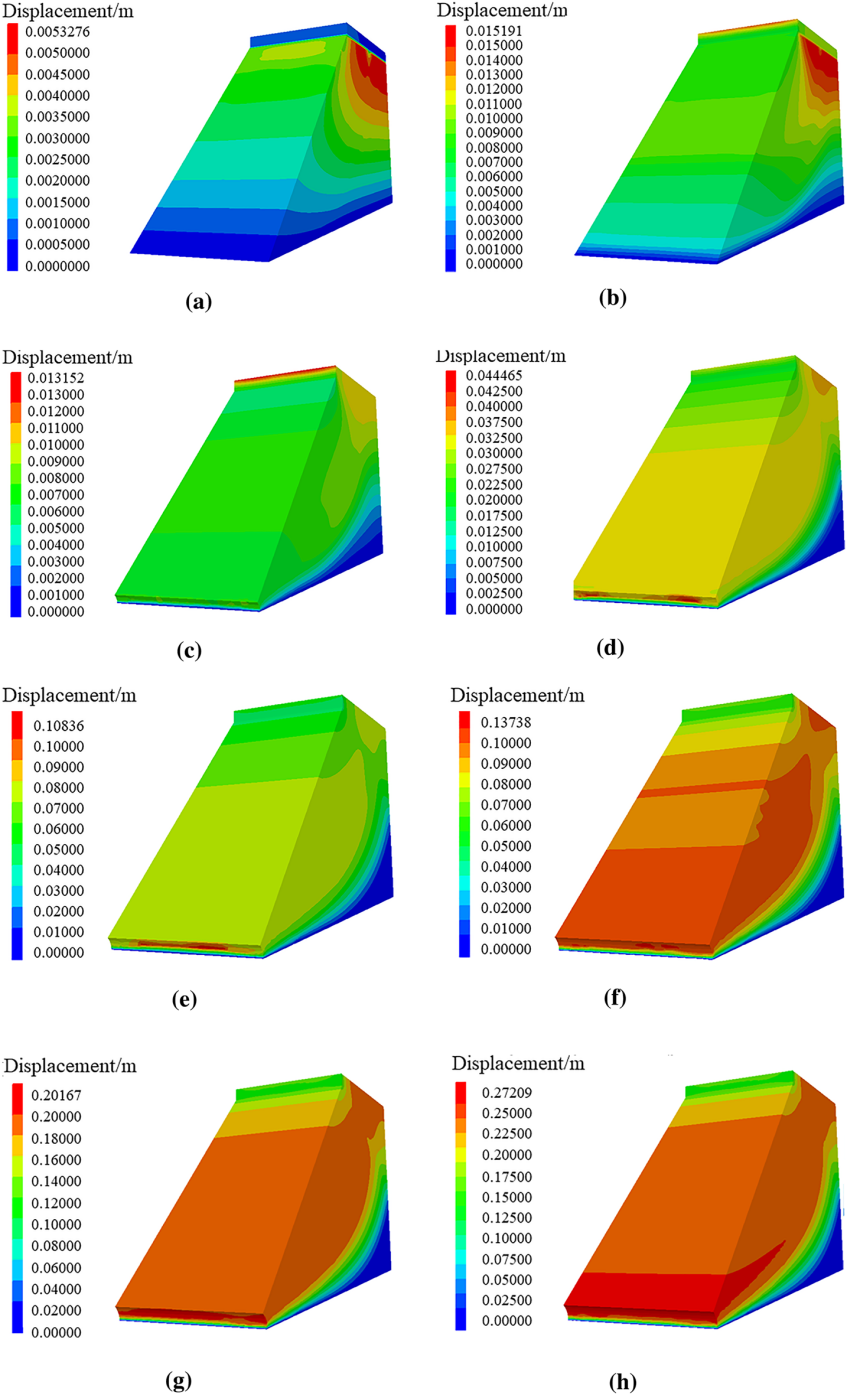

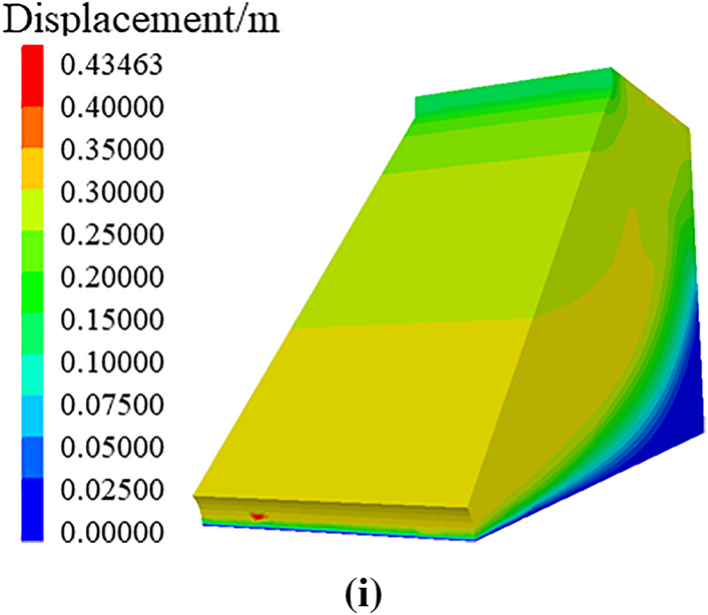
Displacement nephogram of roadbed pavement. (**a**) Displacement cloud image under the action of gravity on subgrade road surface, (**b**) Displacement cloud image under the action of vehicle and gravity, (**c**) Displacement cloud image with water erosion water groove at water depth of 1.6 m, (**d**) Displacement cloud image with erosion groove at water depth of 2.0 m, (**e**) Displacement cloud image with water erosion, groove at water depth of 2.4 m, (**f**) Displacement cloud image with water erosion groove at water depth of 2.8 m, (**g**) Displacement cloud image with water erosion at water depth of 3.2 m (**h**) Displacement cloud image with water groove erosion groove at water depth of 3.6 m, (**i**) Displacement cloud image with water erosion groove at water depth of 4.0 m.

Through numerical simulation of the displacement nephogram of soil roadbed along the river under the condition of only road surface and soil roadbed load, vehicle loading and continuous water depth changes, as shown in Fig. 23, the following change law can be found:

1. Under road surface and soil roadbed load, the displacement nephogram shows that the soil roadbed soil under the road surface is subjected to its gravity, resulting in vertical settlement displacement. With the increase of the soil roadbed depth, the settlement displacement decreases. This is because the soil at the bottom is compacted and the soil at the top is vertically settled.
2. When the vehicle is driving on the road surface, the settlement displacement of roadbed soil under the road surface increases. With the increase of roadbed soil depth, the settlement displacement is higher than that when there is only road surface and roadbed load.
3. When flood scours roadbed soil, the scour displacement at the soil roadbed slope toe changes and water erosion grooves appear.
4. The water erosion groove at the soil roadbed slope toe increases with the increase of flood water level, and the displacement of roadbed soil slope also increases with the increase of water level, which is connected with the soil at the bottom of pavement slab and tends to landslide in the form of sliding surface.

**Figure 24:**
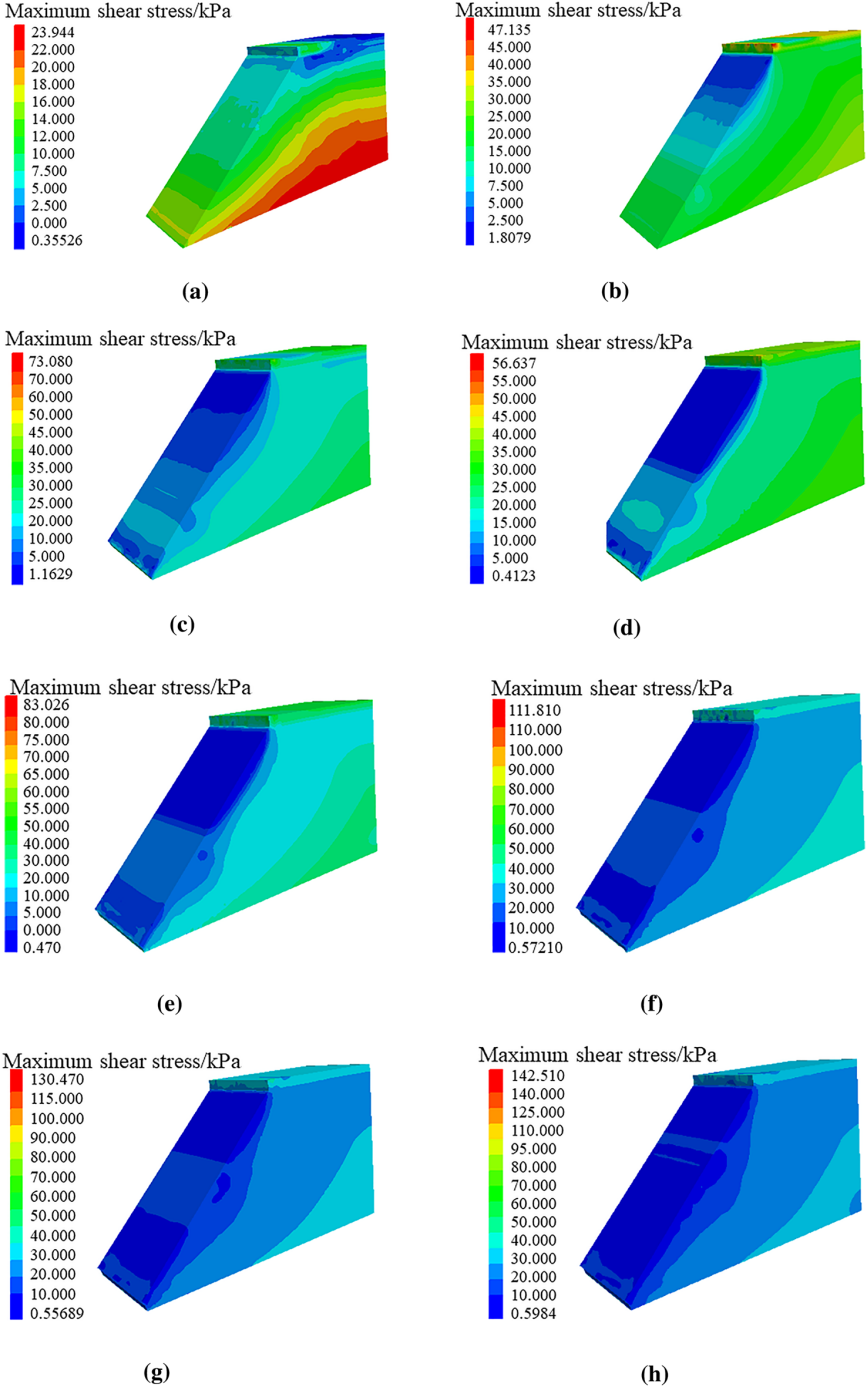

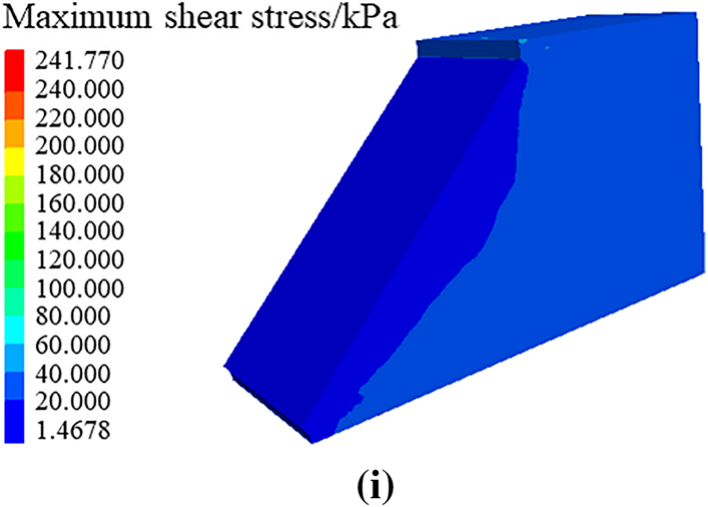
Maximum shear stress nephogram of roadbed and pavement. (**a**) Maximum shear stress nephogram under the action, gravity on subgrade road surface, (**b**) Maximum shear stress nephogram of under the action of vehicle and gravity, (**c**) Maximum shear stress nephogram with water erosion, groove at water depth of 1.6 m (**d**) Maximum shear stress nephogram with water erosion groove at water depth of 2.0 m, (**e**) Maximum shear stress nephogram with water, erosion groove at water depth of 2.4 m (**f**) Maximum shear stress nephogram with, water erosion groove at water depth of 2.8 m, (**g**) Maximum shear stress nephogram with water erosion, groove at water depth of 3.2 m (**h**) Maximum shear stress nephogram with water erosion groove at water depth of 3.6 m, (**i**) Maximum shear stress nephogram with water erosion groove at water depth of 4.0 m.

Through numerical simulation of the maximum shear stress nephograms of soil roadbed along the river under the conditions of only road surface and roadbed load, adding vehicle loading and continuous water depth change, as shown in Fig. 24, the following changing law can be found:

1. When only the road surface and soil roadbed are loaded, the maximum shear stress nephogram shows that the shear stress on the bottom roadbed soil is the largest, and it gradually decreases from bottom to top. This is because the bottom roadbed soil is compacted under the action of gravity.
2. When there are vehicles driving on the road, the shear stress of the road surface is the largest, followed by the overall shear stress of the roadbed soil, which is large and uniform, and the shear stress of the upper part of the roadbed slope is small.
3. When the flood scours roadbed soil, the maximum shear stress at the roadbed slope toe changes, and the water erosion grooves appear.
4. Under the action of maximum shear stress, the stress nephogram of soil roadbed slope changes, there is erosion and loss of roadbed soil. With the increase of water level, the height of loss gradually increases, and the maximum shear stress of roadbed soil changes as a sliding surface.

The displacement and maximum shear stress analysis of the above numerical simulation are consistent with the previous stability analysis conclusion.

## Conclusions

The soil roadbed along the river is eroded by flood at the bottom and falls at the top, which leads to the suspension of the upper pavement slab.

1. By carrying out analysis of theoretical and case calculation, this study obtained the washout development mechanism of soil roadbed along the river, and proposed the process diagram of soil roadbed scouring instability.
2. Based on the flood erosion, the stability law of roadbed along the river was analyzed, the sensitivity analysis of water depth, flow velocity, river bending angle and stability safety factor K under different working conditions was carried out. Also, the sensitivity law of the changes of water depth, flow velocity and river bending angle and the size of residue roadbed along the river after erosion was analyzed.
3. The erosion of roadbed toe along the river is greatly influenced by the direction of water flow, the expansion process of water erosion grooves at the roadbed slope toe is the key factor affecting roadbed collapse and pavement slab suspension along the river, and the water erosion movement at the top of roadbed toe is earlier than that at the bottom.
4. Through numerical simulation, the displacement nephogram and maximum shear stress nephogram of roadbed along the river under gravity, pavement load, vehicle load and water depth change were analyzed. By comparing the above theories with engineering cases, the water damage mechanism of roadbed along the river was further verified.

### Supplementary Information

Supplementary Information.

## Data Availability

All data included in this study are available upon request by contact with the corresponding author.
