# Supplementary material for: Stability analysis of roadbed under flood scouring
Source: Sci Rep. 2024 Feb 21;14:4312. doi: 10.1038/s41598-024-54765-8 (PMC11316754; doi:10.1038/s41598-024-54765-8)
Supplement: Supplementary file 1 — Supplementary Information. [file 41598_2024_54765_MOESM1_ESM.zip › supplementary material/Curve of fixed river bending Angle - fixed velocity - different water depth - b residual, h residual.pdf]

Curve of fixed river bending Angle - fixed velocity - different water depth - b residual, h residual

H2=1.6、2.0、2.4、2.8、3.2、3.6、4.0、4.4、4.8、5.2、5.6、6.0、6.4;
V=1.8、2.0、2.2、2.5、2.8、3.1、3.4、3.7、4.0;
θ=90、105、120、135、150、165、180;
P=2, 3, 5, 8, 10, 15, 20, 25, 30, 35, 40, 45, 50, 55, 60, 65

	Array level	No.	V	H2	θ	br	hr			note
1		1	1.8	1.6	90	7.3549	4.8902			
		2	1.8	2	90	7.3355	4.8775			
		3	1.8	2.4	90	7.3192	4.8668			
		4	1.8	2.8	90	7.3052	4.8576			
		5	1.8	3.2	90	7.2934	4.8497			
		6	1.8	3.6	90	7.2833	4.8431			
		7	1.8	4.0	90	7.2749	4.8375			
	Array level	No.	V	H2	θ	br	hr			note
2		1	1.8	1.6	105	7.4190	4.9329			
		2	1.8	2	105	7.4151	4.9305			
		3	1.8	2.4	105	7.4140	4.9300			
		4	1.8	2.8	105	7.4153	4.9310			
		5	1.8	3.2	105	7.4186	4.9333			
		6	1.8	3.6	105	7.4236	4.9367			
		7	1.8	4.0	105	7.4301	4.9412			
	Array level	No.	V	H2	θ	br	hr			note
3		1	1.8	1.6	120	7.4997	4.9865			
		2	1.8	2	120	7.5152	4.9972			
		3	1.8	2.4	120	7.5334	5.0095			
		4	1.8	2.8	120	7.5538	5.0233			
		5	1.8	3.2	120	7.5761	5.0384			
		6	1.8	3.6	120	7.6001	5.0545			
		7	1.8	4.0	120	7.6255	5.0716			
	Array level	No.	V	H2	θ	br	hr			note
4		1	1.8	1.6	135	7.6032	5.0553			
		2	1.8	2	135	7.5000	5			
		3	1.8	2.4	135	7.5000	5			
		4	1.8	2.8	135	7.5000	5			
		5	1.8	3.2	135	7.5000	5			
		6	1.8	3.6	135	7.5000	5			
		7	1.8	4.0	135	7.8761	5.2387			
	Array level	No.	V	H2	θ	br	hr			note
5		1	1.8	1.6	150	7.5000	5			
		2	1.8	2	150	7.5000	5			
		3	1.8	2.4	150	7.5000	5			
		4	1.8	2.8	150	7.5000	5			
		5	1.8	3.2	150	7.5000	5			
		6	1.8	3.6	150	7.5000	5			
		7	1.8	4.0	150	7.5000	5			

Curve of fixed river bending Angle - fixed velocity - different water depth - b residual, h residual

H2=1.6、2.0、2.4、2.8、3.2、3.6、4.0、4.4、4.8、5.2、5.6、6.0、6.4;
V=1.8、2.0、2.2、2.5、2.8、3.1、3.4、3.7、4.0;
θ=90、105、120、135、150、165、180;
P=2, 3, 5, 8, 10, 15, 20, 25, 30, 35, 40, 45, 50, 55, 60, 65

6	Array level	No.	V	H2	θ	br	hr		note
		1	2	1.6	90	7.3083	4.8593		
		2	2	2	90	7.2778	4.8391		
		3	2	2.4	90	7.2503	4.8209		
		4	2	2.8	90	7.2253	4.8043		
		5	2	3.2	90	7.2025	4.7891		
		6	2	3.6	90	7.1815	4.7752		
		7	2	4.0	90	7.1621	4.7623		
7	Array level	No.	V	H2	θ	br	hr		note
		1	2	1.6	105	7.3741	4.9032		
		2	2	2	105	7.3594	4.8936		
		3	2	2.4	105	7.3477	4.8859		
		4	2	2.8	105	7.3383	4.8797		
		5	2	3.2	105	7.3310	4.8749		
		6	2	3.6	105	7.3254	4.8713		
		7	2	4.0	105	7.3215	4.8687		
8	Array level	No.	V	H2	θ	br	hr		note
		1	2	1.6	120	7.4570	4.9583		
		2	2	2	120	7.4622	4.9620		
		3	2	2.4	120	7.4702	4.9676		
		4	2	2.8	120	7.4805	4.9746		
		5	2	3.2	120	7.4917	4.9829		
		6	2	3.6	120	7.5067	4.9923		
		7	2	4.0	120	7.5221	5.0026		
9	Array level	No.	V	H2	θ	br	hr		note
		1	2	1.6	135	7.5632	5.0289		
		2	2	2	135	7.5940	5.0498		
		3	2	2.4	135	7.6273	5.0723		
		4	2	2.8	135	7.6628	5.0961		
		5	2	3.2	135	7.7001	5.1212		
		6	2	3.6	135	7.7390	5.1473		
		7	2	4.0	135	7.7930	5.1743		
									note

Curve of fixed river bending Angle - fixed velocity - different water depth - b residual, h residual

H2=1.6、2.0、2.4、2.8、3.2、3.6、4.0、4.4、4.8、5.2、5.6、6.0、6.4;
V=1.8、2.0、2.2、2.5、2.8、3.1、3.4、3.7、4.0;
θ=90、105、120、135、150、165、180;
P=2, 3, 5, 8, 10, 15, 20, 25, 30, 35, 40, 45, 50, 55, 60, 65

10	Array level	No.	V	H2	θ	br	hr			note
		1	2.2	1.6	90	7.2651	4.8306			
		2	2.2	2	90	7.2242	4.8305			
		3	2.2	2.4	90	7.1864	4.7784			
		4	2.2	2.8	90	7.1512	4.7549			
		5	2.2	3.2	90	7.1181	4.7329			
		6	2.2	3.6	90	7.0870	4.7121			
		7	2.2	4.0	90	7.0575	4.6925			
11	Array level	No.	V	H2	θ	br	hr			note
		1	2.2	1.6	105	7.3325	4.8756			
		2	2.2	2	105	7.3078	4.8593			
		3	2.2	2.4	105	7.2861	4.8449			
		4	2.2	2.8	105	7.2669	4.8321			
		5	2.2	3.2	105	7.2497	4.8208			
		6	2.2	3.6	105	7.2344	4.8106			
		7	2.2	4.0	105	7.2207	4.8015			
12	Array level	No.	V	H2	θ	br	hr			note
		1	2.2	1.6	120	7.4174	4.9320			
		2	2.2	2	120	7.4131	4.9294			
		3	2.2	2.4	120	7.4116	4.9286			
		4	2.2	2.8	120	7.4125	4.9293			
		5	2.2	3.2	120	7.4154	4.9313			
		6	2.2	3.6	120	7.4200	4.9345			
		7	2.2	4.0	120	7.4262	4.9387			
13	Array level	No.	V	H2	θ	br	hr			note
		1	2.2	1.6	135	7.5262	5.0044			
		2	2.2	2	135	7.5480	5.0193			
		3	2.2	2.4	135	7.5725	5.0359			
		4	2.2	2.8	135	7.5992	5.0538			
		5	2.2	3.2	135	7.6278	5.0730			
		6	2.2	3.6	135	7.6580	5.0933			
		7	2.2	4.0	135	7.6896	5.1145			
14	Array level	No.	V	H2	θ	br	hr			note
		1	2.2	1.6	150	7.6728	5.1018			
		2	2.2	2	150	7.7298	5.1403			
		3	2.2	2.4	150	7.7893	5.1802			
		4	2.2	2.8	150	7.8508	5.2215			
		5	2.2	3.2	150	7.9139	5.2638			
		6	2.2	3.6	150	7.9786	5.3071			
		7	2.2	4.0	150	8.0445	5.3513			

Curve of fixed river bending Angle - fixed velocity - different water depth - b residual, h residual

H2=1.6、2.0、2.4、2.8、3.2、3.6、4.0、4.4、4.8、5.2、5.6、6.0、6.4；
V=1.8、2.0、2.2、2.5、2.8、3.1、3.4、3.7、4.0；
θ=90、105、120、135、150、165、180；
P=2，3，5，8，10，15，20，25，30，35，40，45，50，55，60，65

	Array level	No.	V	H2	θ	br	hr			note
15		1	2.5	1.6	90	7.2055	4.7910			
		2	2.5	2	90	7.1503	4.7543			
		3	2.5	2.4	90	7.0983	4.7196			
		4	2.5	2.8	90	7.0489	4.6867			
		5	2.5	3.2	90	7.0018	4.6553			
		6	2.5	3.6	90	6.9566	4.6252			
		7	2.5	4.0	90	6.9132	4.5962			
	Array level	No.	V	H2	θ	br	hr			note
16		1	2.5	1.6	105	7.2751	4.8374			
		2	2.5	2	105	7.2366	4.8119			
		3	2.5	2.4	105	7.2012	4.7884			
		4	2.5	2.8	105	7.1684	4.7665			
		5	2.5	3.2	105	7.1377	4.7460			
		6	2.5	3.6	105	7.1089	4.7269			
		7	2.5	4.0	105	7.0818	4.7088			
	Array level	No.	V	H2	θ	br	hr			note
17		1	2.5	1.6	120	7.3627	4.8958			
		2	2.5	2	120	7.3453	4.8844			
		3	2.5	2.4	120	7.3308	4.8748			
		4	2.5	2.8	120	7.3187	4.8669			
		5	2.5	3.2	120	7.3087	4.8602			
		6	2.5	3.6	120	7.3005	4.8548			
		7	2.5	4.0	120	7.2939	4.8504			
	Array level	No.	V	H2	θ	br	hr			note
18		1	2.5	1.6	135	7.4751	4.8706			
		2	2.5	2	135	7.4846	4.9772			
		3	2.5	2.4	135	7.4969	4.9856			
		4	2.5	2.8	135	7.5115	4.9955			
		5	2.5	3.2	135	7.5280	5.0066			
		6	2.5	3.6	135	7.5462	5.0188			
		7	2.5	4.0	135	7.5659	5.0320			
	Array level	No.	V	H2	θ	br	hr			note
19		1	2.5	1.6	150	7.6264	5.0712			
		2	2.5	2	150	7.6723	5.1022			
		3	2.5	2.4	150	7.7208	5.1348			
		4	2.5	2.8	150	7.7712	5.1686			
		5	2.5	3.2	150	7.8235	5.2037			
		6	2.5	3.6	150	7.8772	5.2397			
		7	2.5	4.0	150	7.9323	5.2766			

Curve of fixed river bending Angle - fixed velocity - different water depth - b residual, h residual

H2=1.6、2.0、2.4、2.8、3.2、3.6、4.0、4.4、4.8、5.2、5.6、6.0、6.4；
V=1.8、2.0、2.2、2.5、2.8、3.1、3.4、3.7、4.0；
θ=90、105、120、135、150、165、180；
P=2，3，5，8，10，15，20，25，30，35，40，45，50，55，60，65

	Array level	No.	V	H2	θ	br	hr			note
20		1	2.8	1.6	90	7.1510	4.7548			
		2	2.8	2	90	7.0828	4.7093			
		3	2.8	2.4	90	7.0178	4.6660			
		4	2.8	2.8	90	6.9555	4.6244			
		5	2.8	3.2	90	6.8955	4.5844			
		6	2.8	3.6	90	6.8376	4.5457			
		7	2.8	4.0	90	6.7814	4.5081			

	Array level	No.	V	H2	θ	br	hr			note
21		1	2.8	1.6	105	7.2227	4.8026			
		2	2.8	2	105	7.1716	4.7686			
		3	2.8	2.4	105	7.1237	4.7367			
		4	2.8	2.8	105	7.0784	4.7065			
		5	2.8	3.2	105	7.0353	4.6777			
		6	2.8	3.6	105	6.9942	4.6503			
		7	2.8	4.0	105	6.9548	4.6240			

	Array level	No.	V	H2	θ	br	hr			note
22		1	2.8	1.6	120	7.3128	4.8627			
		2	2.8	2	120	7.2834	4.8432			
		3	2.8	2.4	120	7.2570	4.8256			
		4	2.8	2.8	120	7.2331	4.8097			
		5	2.8	3.2	120	7.2113	4.7952			
		6	2.8	3.6	120	7.1913	4.7820			
		7	2.8	4.0	120	7.1730	4.7698			

	Array level	No.	V	H2	θ	br	hr			note
23		1	2.8	1.6	135	7.4284	4.9396			
		2	2.8	2	135	7.4267	4.9387			
		3	2.8	2.4	135	7.4279	4.9397			
		4	2.8	2.8	135	7.4314	4.9421			
		5	2.8	3.2	135	7.4369	4.9459			
		6	2.8	3.6	135	7.4441	4.9507			
		7	2.8	4.0	135	7.4528	4.9566			

										note

Curve of fixed river bending Angle - fixed velocity - different water depth - b residual, h residual

H2=1.6、2.0、2.4、2.8、3.2、3.6、4.0、4.4、4.8、5.2、5.6、6.0、6.4;
V=1.8、2.0、2.2、2.5、2.8、3.1、3.4、3.7、4.0;
θ=90、105、120、135、150、165、180;
P=2, 3, 5, 8, 10, 15, 20, 25, 30, 35, 40, 45, 50, 55, 60, 65

	Array level	No.	V	H2	θ	br	hr	note
24		1	3.1	1.6	90	7.1008	4.7214	
		2	3.1	2	90	7.0205	4.6678	
		3	3.1	2.4	90	6.9435	4.6164	
		4	3.1	2.8	90	6.8693	4.5669	
		5	3.1	3.2	90	6.7975	4.5189	
		6	3.1	3.6	90	6.7277	4.4723	
		7	3.1	4.0	90	6.6598	4.4269	

	Array level	No.	V	H2	θ	br	hr	note
25		1	3.1	1.6	105	7.1743	4.7704	
		2	3.1	2	105	7.1116	4.7286	
		3	3.1	2.4	105	7.0521	4.6890	
		4	3.1	2.8	105	6.9954	4.6511	
		5	3.1	3.2	105	6.9409	4.6147	
		6	3.1	3.6	105	6.8884	4.5797	
		7	3.1	4.0	105	6.8377	4.5458	

	Array level	No.	V	H2	θ	br	hr	note
26		1	3.1	1.6	120	7.2667	4.8320	
		2	3.1	2	120	7.3733	4.9032	
		3	3.1	2.4	120	7.1889	4.7802	
		4	3.1	2.8	120	7.1540	4.7570	
		5	3.1	3.2	120	7.1214	4.7353	
		6	3.1	3.6	120	7.0906	4.7147	
		7	3.1	4.0	120	7.0615	4.6953	

								note

								note

Curve of fixed river bending Angle - fixed velocity - different water depth - b residual, h residual

H2=1.6、2.0、2.4、2.8、3.2、3.6、4.0、4.4、4.8、5.2、5.6、6.0、6.4;
V=1.8、2.0、2.2、2.5、2.8、3.1、3.4、3.7、4.0;
θ=90、105、120、135、150、165、180;
P=2, 3, 5, 8, 10, 15, 20, 25, 30, 35, 40, 45, 50, 55, 60, 65

	Array level	No.	V	H2	θ	br	hr	note
27		1	3.4	1.6	90	7.0541	4.6903	
		2	3.4	2	90	6.9625	4.6291	
		3	3.4	2.4	90	6.8744	4.5703	
		4	3.4	2.8	90	6.7891	4.5133	
		5	3.4	3.2	90	6.7063	4.4580	
		6	3.4	3.6	90	6.6256	4.4041	
		7	3.4	4.0	90	6.5467	4.3514	

	Array level	No.	V	H2	θ	br	hr	note
28		1	3.4	1.6	105	7.1293	4.7404	
		2	3.4	2	105	7.0558	4.6914	
		3	3.4	2.4	105	6.9856	4.6446	
		4	3.4	2.8	105	6.9181	4.5995	
		5	3.4	3.2	105	6.8531	4.5561	
		6	3.4	3.6	105	6.7900	4.5139	
		7	3.4	4.0	105	6.7287	4.4730	

	Array level	No.	V	H2	θ	br	hr	note
29		1	3.4	1.6	120	7.2239	4.8036	
		2	3.4	2	120	7.1731	4.7697	
		3	3.4	2.4	120	7.1255	4.7380	
		4	3.4	2.8	120	7.0805	4.7080	
		5	3.4	3.2	120	7.0378	4.6795	
		6	3.4	3.6	120	6.9969	4.6522	
		7	3.4	4.0	120	6.9578	4.6261	

	Array level	No.	V	H2	θ	br	hr	note
30		1	3.4	1.6	135	7.3452	4.8844	
		2	3.4	2	135	7.3236	4.8701	
		3	3.4	2.4	135	7.3049	4.8578	
		4	3.4	2.8	135	7.2887	4.8470	
		5	3.4	3.2	135	7.2746	4.8376	
		6	3.4	3.6	135	7.2623	4.8294	
		7	3.4	4.0	135	7.2515	4.8223	

								note

Curve of fixed river bending Angle - fixed velocity - different water depth - b residual, h residual

H2=1.6、2.0、2.4、2.8、3.2、3.6、4.0、4.4、4.8、5.2、5.6、6.0、6.4;
V=1.8、2.0、2.2、2.5、2.8、3.1、3.4、3.7、4.0;
θ=90、105、120、135、150、165、180;
P=2, 3, 5, 8, 10, 15, 20, 25, 30, 35, 40, 45, 50, 55, 60, 65

	Array level	No.	V	H2	θ	br	hr			note
31		1	3.7	1.6	90	7.0104	4.6611			
		2	3.7	2	90	6.9083	4.5930			
		3	3.7	2.4	90	6.8098	4.5272			
		4	3.7	2.8	90	6.7141	4.4633			
		5	3.7	3.2	90	6.6210	4.4010			
		6	3.7	3.6	90	6.5300	4.3402			
		7	3.7	4.0	90	6.4409	4.2806			

	Array level	No.	V	H2	θ	br	hr			note
32		1	3.7	1.6	105	7.0872	4.7124			
		2	3.7	2	105	7.0036	4.6566			
		3	3.7	2.4	105	6.9233	4.6030			
		4	3.7	2.8	105	6.8459	4.5513			
		5	3.7	3.2	105	6.7709	4.5012			
		6	3.7	3.6	105	6.6979	4.4524			
		7	3.7	4.0	105	6.6268	4.4049			

	Array level	No.	V	H2	θ	br	hr			note
33		1	3.7	1.6	120	7.1838	4.7769			
		2	3.7	2	120	7.1234	4.7366			
		3	3.7	2.4	120	7.0662	4.6985			
		4	3.7	2.8	120	7.0117	4.6621			
		5	3.7	3.2	120	6.9595	4.6272			
		6	3.7	3.6	120	6.9093	4.5937			
		7	3.7	4.0	120	6.8608	4.5613			

	Array level	No.	V	H2	θ	br	hr			note
34		1	3.7	1.6	135	7.3077	4.8595			
		2	3.7	2	135	7.2771	4.8392			
		3	3.7	2.4	135	7.2495	4.8208			
		4	3.7	2.8	135	7.2244	4.8041			
		5	3.7	3.2	135	7.2014	4.7888			
		6	3.7	3.6	135	7.1803	4.7747			
		7	3.7	4.0	135	7.1608	4.7617			

										note

Curve of fixed river bending Angle - fixed velocity - different water depth - b residual, h residual

H2=1.6、2.0、2.4、2.8、3.2、3.6、4.0、4.4、4.8、5.2、5.6、6.0、6.4;
V=1.8、2.0、2.2、2.5、2.8、3.1、3.4、3.7、4.0;
θ=90、105、120、135、150、165、180;
P=2, 3, 5, 8, 10, 15, 20, 25, 30, 35, 40, 45, 50, 55, 60, 65

	Array level	No.	V	H2	θ	br	hr			note
35		1	4	1.6	90	6.9693	4.6337			
		2	4	2	90	6.8573	4.5589			
		3	4	2.4	90	6.7489	4.4865			
		4	4	2.8	90	6.6436	4.4161			
		5	4	3.2	90	6.5407	4.3474			
		6	4	3.6	90	6.4400	4.2801			
		7	4	4.0	90	6.3413	4.2141			

	Array level	No.	V	H2	θ	br	hr			note
36		1	4	1.6	105	7.0476	4.6860			
		2	4	2	105	6.9544	4.6238			
		3	4	2.4	105	6.8647	4.5639			
		4	4	2.8	105	6.7779	4.5059			
		5	4	3.2	105	6.6935	4.4495			
		6	4	3.6	105	6.6113	4.3945			
		7	4	4.0	105	6.5309	4.3408			

	Array level	No.	V	H2	θ	br	hr			note
37		1	4	1.6	120	7.1461	4.7518			
		2	4	2	120	7.0766	4.7054			
		3	4	2.4	120	7.0105	4.6613			
		4	4	2.8	120	6.9470	4.6189			
		5	4	3.2	120	6.8859	4.5781			
		6	4	3.6	120	6.8268	4.5386			
		7	4	4.0	120	6.7695	4.5003			

	Array level	No.	V	H2	θ	br	hr			note
38		1	4	1.6	135	7.2725	4.8360			
		2	4	2	135	7.2333	4.8100			
		3	4	2.4	135	7.1973	4.7860			
		4	4	2.8	135	7.1638	4.7637			
		5	4	3.2	135	7.1325	4.7428			
		6	4	3.6	135	7.1031	4.7232			
		7	4	4.0	135	7.0754	4.7047			

									note
